# ﻿Taxonomy and phylogeny of entomopathogenic fungi from China—revealing two new genera and thirteen new species within Clavicipitaceae (Hypocreales, Ascomycota)

**DOI:** 10.3897/mycokeys.117.140577

**Published:** 2025-05-05

**Authors:** Zhi-Qin Wang, Zhi-Li Yang, Jing Zhao, Jin-Mei Ma, De-Xiang Tang, Zong-Li Liang, Jian-Hong Li, Xin-Mao Zhou, Hong Yu

**Affiliations:** 1 Yunnan Herbal Laboratory, College of Ecology and Environmental Sciences, Yunnan University, Kunming, Yunnan, China Yunnan University Кunming China; 2 The International Joint Research Centre for Sustainable Utilization of Cordyceps Bioresources in China and Southeast Asia, Yunnan University, Kunming, Yunnan, China Yunnan Jinping Fenshuiling National Nature Reserve Honghe China; 3 Yunnan Jinping Fenshuiling National Nature Reserve, Honghe, Yunnan, China Yunnan University Кunming China; 4 College of Ecology and Environmental Science, Yunnan University, Kunming, Yunnan, China Yunnan Jinping Fenshuiling National Nature Reserve Honghe China

**Keywords:** *Moelleriella*, *Paramoelleriella*, *Polymicrospora*

## Abstract

Scale insects (Coccidae, Hemiptera) and whiteflies (Aleyrodidae, Homoptera) are diminutive, ubiquitous, sap-sucking plant parasites, many of which are serious agricultural pests. Over the course of several years, an investigation into entomopathogenic fungi affecting scale insects and whiteflies resulted in the collection of 13 novel species of Clavicipitaceae in Yunnan and Hainan Provinces, China. Based on three-loci (nrLSU, *tef*-1*a*, and *rpb1*) phylogenetic analysis and morphological evidence, it was determined that two new genera, *Paramoelleriella* and *Polymicrospora*, each encompassed a new species. Additionally, two new species of *Hypocrellas. str.* and nine new species of *Moelleriella* were identified. Within the *Moelleriella* clade, seven new species were assigned to the Effuse clade and two to the Globose clade. *Hypocrella* s. str. and *Samuelsia* were included in the Pulvinate clade, to which the new genus *Paramoelleriella* is closely related, although it forms a distinct branch. *Paramoelleriella* species exhibited characteristics similar to those of *Moelleriella*, including globose to subglobose, yellow to orange teleomorphic stromata, with perithecia densely arranged and fully embedded in the stromatal tissue. Its ascospores disarticulated into short-cylindrical part-spores, and the conidiomata featured large, widely open orifices bearing fusoid conidia curved to one side. Species of the new genus *Polymicrospora* were characterized by thin-pulvinate, snow-white to off-white teleomorphic stromata with surface smooth. These species possessed numerous obpyriform or oval, semi-embedded, and densely arranged perithecia, cylindrical asci, and ascospores that disarticulated into small, oval part-spores in large quantities. This study introduces two new genera and 13 new species, accompanied by detailed illustrations and descriptions.

## ﻿Introduction

The order Hypocreales is capable of parasitizing a wide array of organisms, including plants, arthropods, insects, nematodes, rotifers, other fungi, and immunocompromised humans, making it the most broadly parasitic order of entomopathogenic fungi within the kingdom Fungi (Spatafora et al. 2007; Sung et al. 2007; Kepler et al. 2013, 2017; Lombard et al. 2015; Araújo and Hughes 2016; Wang et al. 2020; Araújo et al. 2022). Within Hypocreales, the family Clavicipitaceae exhibits a broad host range and diverse ecological roles, encompassing saprophytes, symbionts, and pathogens associated with soils, insects, plants, fungi, and other invertebrates (Spatafora et al. 2007; Sung et al. 2007; Kepler et al. 2012; Zhang et al. 2023). To date, the family Clavicipitaceae comprises 56 genera and over 470 species (Hyde et al. 2024).

Members of certain genera serve as valuable biocontrol agents in both natural ecosystems and agricultural settings, including *Pochonia* Bat. & O.M. Fonseca, *Drechmeria* Gams & H.B. Jansson, *Metarhizium* Sorokīn, *Hypocrella* Sacc., and *Moelleriella* Bres (Gams and Zare 2003; Chaverri et al. 2008; Spatafora et al. 2015; Mongkolsamrit et al. 2020). Species within the genera *Pochonia* and *Drechmeria* have shown the most promising biological agents to be developed for the control of plant pathogenic nematodes (Gams and Zare 2003). The members of *Metarhizium* show a global distribution that can infect many insect species, some of which have been used in agriculture and forestry as an environmentally safe alternative to chemical pesticides (Jackson and Jaronski 2008; Ghayedi and Abdollahi 2013; Keyser et al. 2014; Clifton et al. 2018; Beys-da-Silva et al. 2020; Kim et al. 2020; Mongkolsamrit et al. 2020). *Hypocrellas. lato.* species have demonstrated biological control capabilities, particularly in managing scale insects (Coccidae, Homoptera), whiteflies (Aleyrodidae, Homoptera), and mites (Petch 1921; Ferron 1978; Barua 1983; Brady 1984; Ramakers and Samson 1984; Fransen 1987; Rombach and Gillespie 1988; Lourencao et al. 1999; Meekes et al. 2000; Faria and Wraight 2001; Meekes et al. 2002; Homrahud et al. 2016; Qasim et al. 2021). Additionally, other genera within the family Clavicipitaceae, such as *Dussiella* Pat., *Hyperdermium* J.F. White et al., *Helicocollum* Luangsa-ard et al., *Regiocrella* Chaverri & K.T. Hodge, *Orbiocrella* D. Johnson et al., *Conoideocrella* D. Johnson et al., and *Samuelsia* P. Chaverri & K.T. Hodge, have been identified as pathogens affecting scale insects or whiteflies (Sullivan et al. 2000; Chaverri et al. 2005a, b, 2008; Johnson et al. 2009; Luangsa-ard et al. 2017).

Species of *Hypocrellas. lato.* (anamorph *Aschersonia* Mont. *s. lato.*) frequently induce epizootics among scale insects and whiteflies that parasitize living leaves and occasionally branches. The majority of *Hypocrellas. lato.* species are predominantly found in tropical regions, with a limited number occurring in subtropical areas (Petch 1921; Mains 1959a, b; Evans and Hywel-Jones 1990; Hywel-Jones and Evans 1993; Chaverri et al. 2005b, 2008). The genus *Hypocrella* was established by Saccardo (1878) to accommodate four species that were formerly assigned to *Hypocrea* Fr., namely, *Hy.atramentosa* Berk. & M.A. Curtis (now *Myriogenosporaatramentosa* (Berk. & M.A. Curtis) Diehl); *Hy.bambusae* Berk. & Broome (now *Balansiabambusae* (Berk. & Broome) Petch); *Hy.discoidea* Berk. & Broome (now *H.discoidea* (Berk. & Broome) Sacc.); and *Hy.semiamplexa* Berk. (now *Balansia* species) (Hywel-jones NL and Evans HC 1993; Chaverri et al. 2008). Among these, only *H.discoidea* (anamorph *A.samoensis*) remains within the genus *Hypocrella* and serves as its type species. The anamorphs of *Hypocrella* were categorized in the anamorph genus *Aschersonia*, which was established by Montagne in 1848 with *A.tahitensis* Mont. as the type species (Montagne 1848). Previously, it was commonly accepted that the genus *Hypocrella* included ascospores with both disarticulating and non-disarticulating (Petch 1939; Mains 1959a, b; Hywel-Jones and Evans 1993). However, a study conducted by Chaverri et al. (2008) demonstrated that species with disarticulating ascospores formed a monophyletic group and should be segregated from *Hypocrella* and placed in *Moelleriella*.

According to the current classification system, three genera have been separated from *Hypocrellas. lato.*, namely *Hypocrellas. str.* (anamorph *Aschersonias. str.*), *Moelleriella* (anamorph aschersonia-like), and *Samuelsia* P. Chaverri & K.T. Hodge (anamorph aschersonia-like) (Chaverri et al. 2008). Species within *Hypocrellas. str.* were characterized by typically pulvinate, brightly colored stromata; embedded or half-embedded perithecia; cylindrical or clavate asci; filiform to long-fusiform ascospores that do not disarticulate (Chaverri et al. 2008). The characteristics of *Aschersonias. str.* species include pycnidium-like conidiomata, cylindrical phialides, and paraphyses, as well as unicellular, hyaline, fusiform conidia produced in copious slime with brightly colored (Chaverri et al. 2005b, 2008). As of December 15, 2024, the Index Fungorum lists 82 names under *Hypocrella* and 79 names under *Aschersonia* (Index Fungorum: http://www.indexfungorum.org). In 2012, the International Botanical Congress in Melbourne adopted a unitary system of nomenclature, known as One Fungus = One Name (1F = 1N), applicable to all fungi, irrespective of whether they are typified by their teleomorph or anamorph (McNeill et al. 2012). Despite this, many species within the genera *Hypocrella* and *Aschersonia* were initially identified by earlier researchers based on morphological characteristics. The lack of type specimens has hindered subsequent researchers’ ability to link the sexual-type genus *Hypocrella* to the asexual-type genus *Aschersonia* using molecular data. To date, only 23 species of the sexual-type genus *Hypocrella* have been successfully linked to the asexual-type genus *Aschersonia* (Chaverri et al. 2008; Mongkolsamrit et al. 2009).

The genus *Moelleriella* was established to accommodate *M.sulphurea* Bres. (1896) as the type species. Since *M.sulphurea* is currently regarded as a synonym of *M.phyllogena* (Mont.) P. Chaverri & K.T. Hodge (basionym *Hypocrellaphyllogena* (Mont.) Speg.), *M.phyllogena* was consequently designated as the type species of the genus *Moelleriella* (Chaverri et al. 2008). Species within the genus *Moelleriella* were characterized by their predominantly globose, thick pulvinate, tuberculate, convex, or thin pulvinate almost effuse, brightly colored stromata. Perithecia occurs in stroma in three distinct configurations, such as fully embedded, half-embedded, or forming prominent tubercles. These species also exhibited filiform, multiseptate ascospores that disarticulate at the septa within the cylindrical ascus, along with aschersonia-like anamorphs (Chaverri et al. 2008). The anamorphs of *Moelleriella* and *Samuelsia* shared similarities with *Aschersonias. str.*, yet they differed in conidia morphology, with *Moelleriella* possessing fusoid conidia and *Samuelsia* having allantoid conidia (Chaverri et al. 2008). Chaverri et al. (2005b) conducted phylogenetic analyses using DNA sequence data (large subunit nuclear ribosomal DNA (nrLSU), the translation elongation factor 1-α (*tef-1a*), and the largest subunits of RNA polymerase II (*rpb1*) in conjunction with stromatal morphology) to divide the genus *Hypocrellas. lato.* into three clades: the Effuse group clade, the Globose group clade, and the Pulvinate group clade (Chaverri et al. 2005b). Species from the Effuse and Globose clades have since been incorporated into the genus *Moelleriella*. Furthermore, Yang et al. (2023) reported that the Effuse clade should be further subdivided into two sister clades, subclade I and subclade II (Chaverri et al. 2005b, 2008; Yang et al. 2023). At present, subclade I contains 13 species, viz., *M.chiangmaiensis*, *M.flava*, *M.gracilispora*, *M.kanchanaburiensis*, *M.madidiensis*, *M.mollii*, *M.nanensis*, *M.nivea*, *M.ochracea*, *M.phukhiaoensis*, *M.pongdueatensis*, *M.sinensis*, and *M.zhongdongii*; subclade II contains 13 species, viz., *M.alba*, *M.basicystis*, *M.chumphonensis*, *M.disjuncta*, *M.evansii*, *M.libera*, *M.oxystoma*, *M.phyllogena*, *M.puerensis*, *M.raciborskii*, *M.rhombispora*, *M.simaoensis*, and *M.umbospora*; the Globose clade contains nine species, viz., *M.africana*, *M.boliviensis*, *M.epiphylla*, *M.insperata*, *M.macrostroma*, *M.reineckeana*, *M.schizostachyi*, *M.sloaneae*, and *M.turbinata* (Chaverri et al. 2008; Yuan et al. 2020; Chen et al. 2020; Khonsanit et al. 2021; Wang et al. 2022; Yang et al. 2023). At present, the genus *Moelleriella* is known to contain 65 species, of which 30 species are from the New World (including: Belize, Brasilia, Brazil, Bolivia, Canada, Colombia, Costa Rica, Cuba, Ecuador, French Guiana, Grenada, Guadeloupe, Guatemala, Guyana, Honduras, Jamaica, Mexico, Nicaragua, Panama, Peru, Puerto Rico, Saint Vincent, Surinam, Trinidad, USA, Venezuela, and Paraguay) and 35 species from the Old World (including: China, Côte D’Ivoire, Ghana, Indonesia, Thailand, Uganda, Vietnam, and some species unknown due to insufficient data) (Chaverri et al. 2008; Mongkolsamrit et al. 2011, 2015; Li et al. 2016; Tibpromma et al. 2017; Chen et al. 2020; Crous et al. 2020; Yuan et al. 2020; Khonsanit et al. 2021; Wang et al. 2022; Yang et al. 2023). Only four new species have been reported in China, of which two are located in Yunnan Province (*M.simaoensis* Hong Yu bis, Z. L. Yang & Z. Q. Wang, and *M.puerensis* Hong Yu bis et al.), and the other two are located in Fujian Province (*M.sinensis* Jun Z. Qiu & Y.X. Chen and *M.gracilispora* Jun Z. Qiu & Y.X. Chen) (Chen et al. 2020; Yuan et al. 2020; Wang et al. 2022; Yang et al. 2023).

Over the past two decades, our research group has undertaken extensive surveys and specimen collections of entomopathogenic fungi across China. In this study, 40 specimens were collected from field surveys conducted in Yunnan and Hainan Provinces. These specimens were analyzed using morphological characteristics combined with a multi-gene phylogeny based on the Bayesian inference (BI) and the maximum likelihood (ML) methods (using nrLSU, *rpb*1, and *tef*-1*a* loci). Our findings confirmed the presence of two new genera (*Paramoelleriella* and *Polymicrospora*), nine new species of *Moelleriella*, and two new species of *Hypocrella*.

## ﻿Materials and methods

### ﻿Fungal collection and isolation

In this study, fungus-infected scale insects and whitefly specimens attached to the upper side and underside of leaves were collected from Yunnan and Hainan Provinces in China. Most of the specimens were sourced from three cities within Yunnan Province, namely Pu’er, Jinghong, and Baoshan, as well as Yuanyang and Jingping County. Some specimens were also obtained from Haikou City, Changjiang County, and Qiongzhong County in Hainan Province. In the field, detailed notes were taken regarding the vegetation type, living host species, and the specific location of stromata on the leaves. Subsequently, entire leaves, where stromata were predominantly found on living leaves but occasionally on leaf litter, were carefully placed in sterilized plastic containers and transported to the laboratory for further processing. To establish axenic cultures, the stromata were excised from the leaves and subjected to a 30-second soak in a 30% hydrogen peroxide solution, with the duration adjusted based on the size of the stromata. This was followed by a 60-second rinse in sterile water. The stromata were then transferred to sterile filter paper, sectioned into 2–4 pieces using sterile dissecting knives, dried on sterile filter paper, and finally inoculated onto potato dextrose agar (PDA) plates supplemented with 0.1 g/L streptomycin and 0.05 g/L tetracycline. The pure cultures were incubated at room temperature (approximately 25 °C). Following successful isolation, the cultures were transferred to PDA slants and stored at 4 °C.

### ﻿Preserving and maintaining specimens and cultures

The specimens have been deposited in the Yunnan Herbal Herbarium (YHH) located in the Yunnan Herbal Laboratory. Additionally, the strain has been deposited in the Yunnan Fungal Culture Collection (YFCC), also situated within Yunnan Herbal Laboratory.

### ﻿Morphological characterization

Macro-morphological characteristics of the stroma, including size, color, shape, and hardness, were quantitatively assessed under a dissecting microscope (SZ61, Olympus Corporation, Tokyo, Japan) following the methodology outlined by Chaverri et al. (2008). Stromata were sectioned to a precise thickness of 40 µm using a Freezing Microtome HM525NX (Thermo Fisher Scientific, Massachusetts, USA). The prepared sections were subsequently mounted on slides with either water or lactic acid-cotton blue. Observations, measurements, and photographic documentation of the perithecia, asci, ascospores, pycnidia, paraphyses, phialides, and conidia were conducted using a light microscope (Olympus BX53). Cultures were grown on PDA for three weeks at 25 °C in an incubator, after which morphological features were captured using a Canon 750D camera (Canon Inc., Tokyo, Japan). Anamorphic structures in culture were also examined under a light microscope (Olympus BX53). The growth rates of the colonies were categorized based on the criteria outlined by Liu and Hodge (2005), which included fast-growing colonies (30–35 mm in diameter), moderately growing colonies (20–30 mm in diameter), and slow-growing colonies (<20 mm in diameter).

### ﻿DNA extraction, PCR amplification, and sequencing

Genomic DNA was extracted from the specimens utilizing the Genomic DNA Purification Kit (Qiagen GmbH, Hilden, Germany) according to the manufacturer’s protocol. In the case of axenic living cultures, DNA extraction was conducted using the CTAB method, as outlined by Liu et al. (2001). Sequencing was conducted on three genes: nuclear ribosomal large subunit (nrLSU), translation elongation factor 1α (*tef*-1*α*), and largest subunits of RNA polymerase II (*rpb*1). The following primer pairs were utilized for PCR amplification: nrLSU was amplified using the primer pairs LR5 and LR0R (Vilgalys and Hester 1990; Rehner and Samuels 1994); *tef*-1*α* was amplified with the primers EF1α-EF and EF1α-ER (Bischof et al. 2006; Sung et al. 2007); and *rpb*1 was amplified with the primers RPB1-5’F and RPB1-5’R (Bischof et al. 2006; Sung et al. 2007. These primer pairs were synthesized by Kunming Xiuqi Technology Co., Ltd. The PCR reactions were carried out in a total volume of 50 µL, comprising 25 µL of 2× Taq PCR Master Mix (Tiangen Biotech Co., Ltd., Beijing, China), 0.5 µL of each forward and reverse primer (10 µM), 1 µL of genomic DNA, and 23 µL of sterile distilled water. The amplification reactions were executed in a BIORAD T100™ thermal cycler (BIO-RAD Laboratories, Hercules, CA, USA), following the procedures detailed by Wang et al. (2015). Subsequently, the PCR products were sequenced at the Beijing Genomics Institute (Chongqing, China).

### ﻿Phylogenetic analyses

The DNA sequences generated in this study, including three genes (nrLSU, *tef*‐1*α*, and *rpb*1), were obtained from 40 samples of 13 species that belonged to four genera and submitted to GenBank. Phylogenetic analyses based on the three genes were performed using datasets retrieved from GenBank and combined with those generated in our study. The taxon information and GenBank accession numbers were provided in Table 1, and most sequences that were downloaded from the GenBank database were based on previous studies by Mongkolsamrit et al. (2009), Luangsa-ard et al. (2017), Khonsanit et al. (2021), and Zhang et al. (2023). Sequences were aligned using Clustal X v.2.0 (developed by the European Bioinformatics Institute, Cambridge, UK), and MEGA v.6.06 (developed by Tokyo Metropolitan University, Tokyo, Japan) was used to remove poorly aligned regions and for manual adjustment (Larkin et al. 2007; Tamura et al. 2013). After sequence alignment and specific processing, according to Wang et al. (2020), the aligned sequences of the three genes were concatenated. Phylogenetic analyses of the dataset were performed using the Bayesian inference (BI) and maximum likelihood (ML) methods, which employ MrBayes v.3.2.2 and IQ-tree v.2.1.3, respectively (Ronquist et al. 2012; Nguyen et al. 2015). The best-fitting likelihood model for BI and ML analyses was selected using Modelfinder (Kalyaanamoorthy et al. 2017). The best nucleotide evolution model was chosen based on the Akaike information criterion (AIC). The TIM2+F+I+G4 model was selected as the optimal model for the ML analyses, with 5000 ultrafast bootstraps (Hoang et al. 2017) in a single run. The GTR+F+I+G4 model was selected as the optimal model for the BI analysis. The four Markov Chain Monte Carlo chains run for 2 million generations from a random start tree with a sampling frequency of 100 generations, and the first 25% of samples were discarded as burn-in. Phylogenetic trees were visualized in Figtree (v.1.4.3) and edited in Adobe Illustrator CS6.

**Table 1.** Names, voucher information, and corresponding GenBank accession numbers of the taxa used in this study.

Species	Strain	Origin	GenBank accession numbers		
			nrLSU	*tef*–1*α*	*rpb*1
*Aciculosporiumoplismeni*	MAFF 246966	Japan	LC571760	LC572040	–
*A.take*	MAFF 241224	Japan	LC571753	LC572034	–
*A.take*	TNS: F-60469	Japan	LC571756	LC572035	–
*Albacilliumhingganense*	SGSF 339	China	OR740566	MN065771	OR769082
*Atkinsonellabypoxylon*	B4728	–	–	KP689546	–
*Balansiahenningsiana*	AEG96-27a	USA	AY489715	AY489610	AY489643
*Clavicepsfusiformis*	ATCC 26019	Zimbabwe	U17402	DQ522320	DQ522366
*C.purpurea*	GAM 12885	Germany	AF543789	AF543778	AY489648
*C.purpurea*	SA cp11	Germany	EF469075	EF469058	EF469087
*C.paspali*	ATCC 13892	USA	U47826	DQ522321	DQ522367
*Collarinaaurantiaca*	CBS:138274	Spain	OR052104	–	–
*C.aurantiaca*	CBS110646	Netherlands	OQ055447	OQ470828	–
*Commelinaceomycesaneilematis*	CBS110646	Japan	LC474617	LC474623	LC474626
*Conoideocrellaluteorostrata*	NHJ 11343	Thailand	EF468850	–	EF468906
*C.luteorostrata*	NHJ 12516	Thailand	EF468849	EF468800	EF468905
*C.tenuis*	NHJ 6293	Thailand	EU369044	EU369029	EU369068
*C.tenuis*	NHJ 6791	Thailand	EU369046	EU369028	EU369069
*Corallocytostromaornithocopreoides*	WAC 8705	Western Australia	–	LT216546	–
*Dussiellatuberiformis*	ATCC 201937	USA	JQ257009	–	JQ257015
*Epbelistripsaci*	CBS 857.72	–	NG_059240	–	–
*Epichloetyphina*	ATCC 56429	Santa Cruz	U17396	AF543777	AY489653
*E.elymi*	C. Schardl760	USA	AY986924	AY986951	–
*Helicocollumsurathaniensis*	BCC34463	Thailand	KT222328	KT222336	–
*H.surathaniensis*	BCC34464	Thailand	KT222329	KT222337	–
*Heteroepichloebambusae*	–	China	MK691595	–	–
*Hypocrellacalendulina*	BCC20309	Thailand	GU552154	–	–
*H.citrina*	P.C. 597	Bolivia	AY986905	AY986930	–
H.cfdiscoidea	I93-901D	Côte D’Ivoire	EU392567	EU392646	EU392700
H.cfdiscoidea	I95-901D	Côte D’Ivoire	EU392568	EU392647	EU392701
*H.discoidea*	BCC 2097	Thailand	–	AY986945	DQ000346
*H.disciformis*	P.C. 655	Honduras	EU392560	EU392643	EU392697
*H.disciformis*	P.C. 676	Honduras	EU392566	EU392645	EU392699
*H.hirsuta*	P.C. 436.2	Mexico	AY986922	AY986949	DQ000350
*H.hirsuta*	P.C. 543	Bolivia	EU392569	EU392648	EU392702
***H.limushanensis***	**YHH 2303015**	**China**	**OR828401**	**OR832089**	**OR837107**
***H.limushanensis***	**YHH 2303016**	**China**	**OR828402**	**OR832090**	**OR837108**
*H.viridans*	P.C. 635	Honduras	EU392572	EU392651	EU392705
*H.viridans*	P.C. 670	Honduras	EU392574	EU392652	EU392706
***H.yunnanensis***	**YHH 2305020**	**China**	**OR828417**	**OR854260**	**OR837109**
***H.yunnanensis***	**YHH 2305021**	**China**	–	**OR854261**	**OR837110**
*Keithomycescarneus*	CBS 239.32	France	NG_057769	EF468789	EF468894
*K.aciculare*	FKI-7236	Japan	LC435741	LC462188	–
*K.aciculare*	FKI-7513	Japan	LC435742	LC462189	–
*Marquandomycesmarquandii*	CBS 182.27	Channel Islands	EF468845	EF468793	EF468899
*M.marquandii*	CBS 128893	Channel Islands	MH876582	–	–
*Metapochoniabulbillosa*	FKI-4395	Denmark	AB709809	AB758460	AB758663
*M.bulbillosa*	CBS 145.70	Denmark	AF339542	EF468796	EF468902
*Metarhiziopsismicrospora*	INEHS133a	USA	EF464572	–	–
*Metarhiziumflavoviride*	CBS 125.65	Thailand	MT078854	MT078846	MT078862
*M.flavoviride*	CBS 700.74	Thailand	MT078855	MT078847	MT078863
*M.album*	ARSEF 2082	Sri Lanka	DQ518775	DQ522352	KJ398617
*M.anisopliae*	BUM_1900	Tasmania	MH143820	MH143854	MH143869
*M.baoshanense*	CCTCCM2016589	China	KY264174	KY264169	KY264180
*M.baoshanense*	BUM63.4	China	KY264175	KY264170	KY264181
*Moelleriellaafricana*	P.C. 736	Ghana	AY986917	AY986943	DQ000344
*M.alba*	BCC49409	Thailand	JQ269646	KX254423	JQ256906
*M.alba*	BCC49492	Thailand	JQ269645	KX254424	JQ256905
*M.boliviensis*	P.C.603	Bolivia	AY986923	AY986950	DQ000351
*M.basicystis*	F183147	Panama	EU392577	EU392653	–
*M.basicystis*	P.C.374	Costa Rica	AY986903	AY986928	DQ000329
*M.chiangmaiensis*	BCC18029	Thailand	MT659360	MW091560	–
*M.chiangmaiensis*	BBH33051	Thailand	MT659362	MT672277	MT672269
*M.chiangmaiensis*	BCC60941	Thailand	MT659361	MT672278	MT672270
*M.chumphonensis*	BCC47574	Thailand	JQ269647	KX254421	JQ256907
*M.chumphonensis*	BBC47575	Thailand	JQ269648	KX254422	JQ256908
*M.disjuncta*	J.B.205	Panama	EU392578	EU392654	–
*M.epiphylla*	P.C.545	Bolivia	EU392585	EU392660	EU392711
*M.epiphylla*	I93-813	Guiana	EU392583	EU392656	EU392707
*M.evansii*	P.C.627	Ecuador	AY986916	AY986942	DQ000343
*M.flava*	BCC60924	Thailand	KF951146	KX254430	MT672271
*M.flava*	BCC60925	Thailand	KF951147	KX254431	MT672272
*M.flava*	BCC60929	Thailand	KX298238	KX254432	MT672273
*M.gracilispora*	CGMCC3.18989	China	KC964202	KC964191	KC964179
*M.gracilispora*	CGMCC3.18990	China	KC964203	KC964192	KC964180
***M.globostromata***	**YFCC 22109275**	**China**	**OR828408**	**OR831942**	**OR831952**
***M.globostromata***	**YHH 221009**	**China**	–	**OR831941**	**OR831951**
***M.globostromata***	**YHH 221010**	**China**	**OR828403**	**OR831940**	**OR831950**
***M.hainanensis***	**YHH 2303020**	**China**	**OR828400**	**OR831938**	**OR831948**
***M.hainanensis***	**YFCC 23039277**	**China**	–	**OR831939**	**OR831949**
*M.insperata*	ARSEF 2396	Philippines	AY518374	DQ070029	EU392713
***M.jinghongensis***	**YFCC 23089312**	**China**	–	**OR854253**	**OR837093**
***M.jinghongensis***	**YHH 2308025**	**China**	**OR828411**	**OR854254**	**OR837094**
***M.jinghongensis***	**YHH 2308026**	**China**	**OR828409**	**OR854255**	**OR837095**
***M.jinghongensis***	**YHH 2308028**	**China**	**OR828410**	**OR854256**	**OR837096**
*M.kanchanaburiensis*	BCC75979	Thailand	MT659363	MT672279	MT843900
*M.kanchanaburiensis*	BCC75980	Thailand	MT659364	MT672280	MT843901
*M.kanchanaburiensis*	BCC75981	Thailand	MT659365	MT672281	–
*M.libera*	P.C. 444	Mexico	EU392591	EU392662	EU392714
*M.libera*	P.C. 445	Mexico	AY986900	AY986925	DQ000326
*M.macrostroma*	J.B. 115	Costa Rica	AY986920	AY986947	DQ000348
*M.macrostroma*	P.C. 605	Bolivia	AY986919	AY986946	DQ000347
*M.madidiensis*	P.C. 569	Bolivia	AY986915	AY986941	DQ000342
*M.madidiensis*	P.C. 594	Bolivia	EU392595	EU392666	EU392718
*M.mollii*	I93-901A	Côte D’Ivoire	EU392599	EU392667	EU392719
*M.mollii*	I93-901C	Côte D’Ivoire	EU392600	EU392668	EU392720
***M.multiperitheciata***	**YFCC 23089307**	**China**	**OR828407**	**OR832085**	**OR837089**
***M.multiperitheciata***	**YFCC 22109308**	**China**	**OR828406**	**OR832086**	**OR837090**
***M.multiperitheciata***	**YHH 2308010**	**China**	–	**OR832087**	**OR837091**
***M.multiperitheciata***	**YHH 2308011**	**China**	–	**OR832088**	**OR837092**
*M.nanensis*	BCC66303	Thailand	KX298236	KX254427	MW085940
*M.nanensis*	BCC66305	Thailand	MW080317	KX254428	MW085941
*M.nivea*	BCC60891	Thailand	MW080318	MT672282	MW085942
*M.nivea*	BCC58543	Thailand	MT659366	MT672283	MT672274
*M.nivea*	BCC58544	Thailand	MT659367	MT672284	MT843898
*M.ochracea*	P.C. 626	Ecuador	EU392604	EU392670	EU392722
*M.ochracea*	IE 1308	Mexico	EU392601	EU392669	EU392721
*M.phukhiaoensis*	BCC 19769	Thailand	KT880502	–	KT880506
*M.phukhiaoensis*	BCC 19773	Thailand	KT880503	–	KT880507
***M.pseudothanathonensis***	**YFCC 22099302**	**China**	–	**OR842379**	**OR837103**
***M.pseudothanathonensis***	**YFCC 22099303**	**China**	–	**OR842380**	**OR837104**
***M.pseudothanathonensis***	**YHH 2209004**	**China**	–	**OR842381**	**OR837105**
***M.pseudothanathonensis***	**YHH 2209005**	**China**	**OR828404**	**OR842382**	**OR837106**
*M.phyllogena*	P.C. 555	Bolivia	EU392610	EU392674	EU392726
*M.phyllogena*	J.B. 130	Panama	EU392608	EU392672	EU392724
*M.pongdueatensis*	BCC31787	Thailand	KT880500	KX254433	KT880504
*M.pongdueatensis*	BCC31788	Thailand	KT880501	KX254434	KT880505
*M.puerensis*	YFCC 8615	China	MW786748	MW815596	MW815595
*M.puerensis*	YFCC 8626	China	MW786750	MW815598	MW815594
***M.puwenensis***	**YHH 2308029**	**China**	**OR828412**	**OR854257**	**OR831953**
***M.puwenensis***	**YHH 2308030**	**China**	**OR828413**	**OR854258**	**OR831954**
***M.puwenensis***	**YHH 2308031**	**China**	**OR828414**	**OR854259**	**OR831955**
***M.qionzhongensis***	**YHH 2303021**	**China**	**OR828399**	**OR831936**	**OR831946**
***M.qionzhongensis***	**YFCC 23039306**	**China**	–	**OR831937**	**OR831947**
*M.raciborskii*	Afr 28	Ghana	DQ070113	EU392675	EU392727
*M.raciborskii*	I93-901b	Côte D’Ivoire	EU392611	EU392676	EU392728
*M.rhombispora*	P.C. 467	Costa Rica	AY986908	AY986933	DQ000334
*M.rhombispora*	P.C. 696	Honduras	EU392618	EU392680	EU392732
*M.schizostachyi*	CBS 100067	Thailand	AY986921	AY986948	DQ000349
*M.simaoensis*	YHH 2210015	China	OQ621807	OQ623179	OQ616915
*M.simaoensis*	YHH 2210016	China	OQ621808	OQ623180	OQ616916
*M.sinensis*	BCC69128	Thailand	KX298234	KX254425	MT843899
*M.sinensis*	BCC69129	Thailand	KX298235	KX254426	MT672275
*M.sinensis*	CGMCC3.18911	China	MK412091	–	MK412101
*M.sloaneae*	I94-920	Guatemala	EU392621	EU392682	EU392734
*M.sloaneae*	I94-922C	Belize	EU392622	EU392683	EU392735
*M.thanathonensis*	MFLU 16 2922	Thailand	–	KY646200	–
*M.turbinata*	IMI 352838	Mexico	EU392625	EU392685	EU392737
*M.turbinata*	P.C. 678	Honduras	EU392627	EU392687	EU392739
*M.umbospora*	P.C. 461	Mexico	EU392628	EU392688	EU392740
*M.umbospora*	P.C. 457	Mexico	AY986904	AY986929	DQ000330
***M.yuanyangensis***	**YFCC 23039314**	**China**	**OR828505**	**OR831945**	**OR837097**
***M.yuanyangensis***	**YHH 2209001**	**China**	–	**OR831944**	**OR837098**
***M.yuanyangensis***	**YHH 2209002**	**China**	–	**OR831943**	**OR837099**
***M.yunnanensis***	**YFCC 23089310**	**China**	–	**OR832093**	**OR837102**
***M.yunnanensis***	**YHH 2308001**	**China**	**OR828416**	**OR832091**	**OR837100**
***M.yunnanensis***	**YHH 2308002**	**China**	**OR828415**	**OR832092**	**OR837101**
*M.zhongdongii*	P.C. 504	Costa Rica	EU392631	EU392689	EU392741
*M.zhongdongii*	P.C. 549	Bolivia	EU392632	EU392690	EU392742
*Mycophilomycespericoniae*	CPC 27558	Malaysia	CPC 27558	–	–
*Myriogenosporaatramentosa*	AEG96-32	Cuba	AY489733	AY489628	AY489665
*Metacordycepschlamydosporia*	JCM18603	Japan	–	AB758464	AB758667
*M.chlamydosporia*	JCM18608	Japan	–	AB758481	AB758684
*Neobaryaparasitia*	Marson s/n	Luxembourg	KP899626	–	–
*Neoaraneomycesaraneicola*	DY101711	China	MW730609	MW753033	–
*N.araneicola*	DY101712	China	MW730610	MW753034	–
*Nigeliaaurantiaca*	BCC 37621	Thailand	GU979946	GU979955	GU979964
*N.aurantiaca*	BCC 13019	Thailand	GU979948	GU979957	–
*N.martiale*	HMAS 197472(S)	Sao Paulo	JF415973	JF416015	JN049892
*N.martialis*	EFCC 6863	Sao Paulo	JF415974	JF416016	–
*Nigrocornusscleroticus*	LB2015_09-18/3	Benin	MK660215	MN104682	–
*Orbiocrellapetchii*	NHJ 5318	Thailand	EU369040	EU369021	EU369062
*O.petchii*	NHJ 6209	Thailand	EU369039	EU369023	EU369061
*Parametarbiziumchangbaiense*	SGSF125	China	MN589994	MN908589	–
*P.hingganense*	SGSF35	China	MN061635	MN065770	–
***Paramoelleriellacurvospora***	**YHH 2305001**	**China**	–	**OR854262**	**OR837111**
***P.curvospora***	**YHH 2305002**	**China**	**OR835994**	**OR854263**	**OR837112**
*Paraneoaraneomycessinensis*	ZY 22.006	China	OQ709260	OQ719626	–
*P.sinensis*	ZY 22.007	China	OQ709261	OQ719627	–
*P.sinensis*	ZY 22.008	China	OQ709262	OQ719628	–
*Papiliomycesshibinensis*	GZUHSB13050311	China	–	KR153589	KR153590
*Periglandulaipomoeae*	IasaF13	Ecuador	–	–	–
*Petchiasiamensis*	BCC 73636	Thailand	MK632089	MK632060	–
*P.siamensis*	BCC68421	Thailand	MK632088	MK632059	MK632164
*P.siamensis*	BCC68420	Thailand	MK632087	–	MK632163
*Pleurocordycepsaurantiaca*	MFLUCC 17- 2113	Thailand	MG136910	MG136875	MG136866
*P.marginaliradians*	MFLU 17-1582	Thailand	–	MG136878	MG136869
*Pochoniasinensis*	ZY 22.009	China	OQ709263	OQ719629	–
*P.sinensis*	ZY 22.010	China	OQ709264	OQ719630	–
***Polymicrosporacaiyangheensis***	**YHH 2309001**	**China**	**OR835995**	**OR854247**	**OR837113**
***P.caiyangheensis***	**YHH 2309002**	**China**	**OR835996**	**OR854248**	**OR837114**
***P.caiyangheensis***	**YHH 2309003**	**China**	**OR835997**	**OR854249**	**OR837115**
***P.caiyangheensis***	**YHH 2309004**	**China**	**OR835998**	**OR854250**	**OR837116**
***P.caiyangheensis***	**YHH 2309005**	**China**	**OR835999**	**OR854251**	–
***P.caiyangheensis***	**YHH 2309006**	**China**	**OR836000**	**OR854252**	**OR837117**
*Pseudometarbiziumaraneogenum*	DY101741	China	MW730618	MW753037	–
*P.araneogenum*	DY101801	China	MW730623	MW753039	–
*Purpureocilliumlavendulum*	FMR 10376	Venezuela	FR775489	FR775516	FR775512
*P.lilacinum*	CBS 284.36	–	FR775484	FR734156	FR775507
*P.lilacinum*	CBS 431.87	–	EF468844	EF468791	EF468897
*Purpureomyceskhaoyaiensis*	BCC1376	Thailand	KX983462	KX983457	–
*P.maesotensis*	BCC88441	Thailand	MN781877	MN781734	–
*Regiocrellacamerunensis*	ARSEF 7682	Cameroon	DQ118735	DQ118743	DQ127234
*R.sinensis*	CUP-CH 2640	Guangdong	DQ118736	DQ118744	DQ127235
*Rotiferophthoraangustispora*	CBS 101437	Rotifera	AF339535	AF543776	DQ522402
*Samuelsiageonomis*	P.C. 614	Bolivia	EU392638	EU392692	EU392744
*S.sheikhii*	P.C. 686	Honduras	EU392639	EU392693	EU392745
*S.chalalensis*	P.C. 560	Bolivia	EU392637	EU392691	EU392743
*S.mundiveteris*	BCC40021	Thailand	GU552152	GU552145	–
*S.mundiveteris*	BCC40022	Thailand	GU552153	GU552146	–
*S.rufobrunneaS*	P.C. 613	Bolivia	AY986918	AY986944	–
*Shimizuomycesparadoxus*	EFCC 6279	Japan	EF469084	EF469071	EF469100
*S.paradoxus*	EFCC 6564	Japan	EF469083	EF469072	EF469101
*Subuliphorumcamptosporum*	CBS 756.69	Germany	OQ430129	OQ471210	
*S.camptosporum*	CBS 757.69	–	OQ430130	OQ471211	–
*S.camptosporum*	CBS:835.91	Cuba	OQ430128	OQ471209	–
*Sungiayongmumensis*	EFCC 2131	–	EF468833	EF468770	EF468876
*S.yongmumensis*	EFCC 2135	–	EF468834	EF468769	EF468877
*Tyrannicordycepsfratricida*	TNS 19011	–	JQ257023	JQ257028	–
*Ustilaginoideadichromonae*	MRLIB 9228	–	–	JQ257025	–
*U.virens*	ATCC 16180	Tamil Nadu	–	JQ257026	–
*U.virens*	MAFF 240421	Tamil Nadu	JQ257011	JQ257024	–
*Yosiokobayasiakusanagiensis*	TNS-F 18494	Japan	JF415972	JF416014	JN049890
*Verticilliumepiphytum*	CBS 384.81	Japan	AF339547	DQ522361	DQ522409

New species were shown in bold.

## ﻿Results

### ﻿Phylogenetic analyses

A dataset comprising 213 strains, including 50 genera of Clavicipitaceae and one genus of Ophiocordycipitaceae, was used for the ML and the BI phylogenetic analyses. The use of data mainly refers to the studies of Chen et al. (2022), Zhang et al. (2023), and Hyde et al. (2024). Two species of *Pleurocordyceps* (*P.aurantiaca* MFLUCC 17-2113 and *P.marginaliradians* MFLU 17-1582) were designated as outgroups. The length of the concatenated three-gene sequence dataset was 2,978 bp, including 1,047 bp for nrLSU, 1,038 bp for *tef*-1*α*, and 893 bp for *rpb*1. Both phylogenetic trees constructed using the ML and the BI analyses were identical in overall topologies and strongly supported in most branches.

The newly established genus *Paramoelleriella* has been classified alongside *Hypocrellas. str.* and *Samuelsia*. Within this clade, the current study identifies three novel species, namely *H.limushanensis*, *H.yunnanensis*, and *P.curvospora*, in addition to seven and five previously recognized species of *Hypocrellas. str.* and *Samuelsia*, respectively. Furthermore, a second new genus, *Polymicrospora*, has been found to cluster with *Collarina*, encompassing one new species, namely *Polymicrosporacaiyangheensis*.

**Figure 1.**
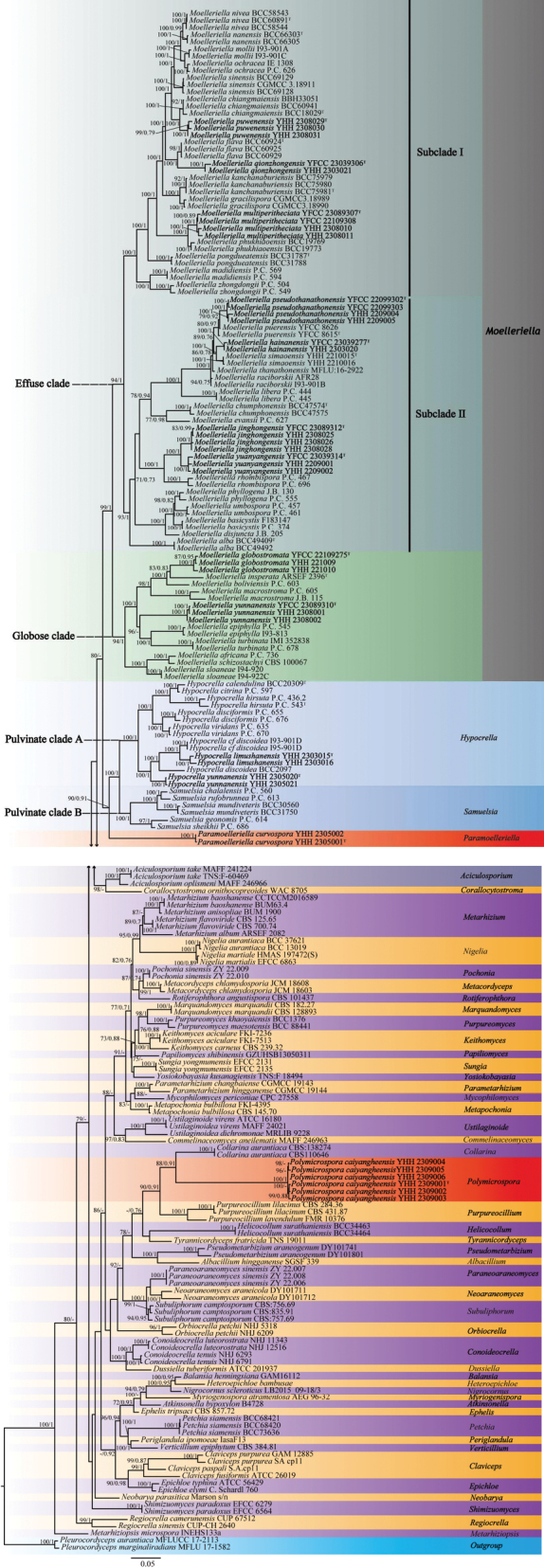
Phylogenetic relationships of Clavicipitaceae based on the maximum likelihood (ML) and Bayesian inference (BI) analysis using three-gene (nrLSU, *tef*-1*α*, and *rpb*1) sequences. The values of the ML bootstrap proportions (≥ 70%) and the BI posterior probability (≥ 0.70) are indicated at the nodes (BP/PP). The new taxa are highlighted in bold. Isolates representing ex-type material are marked with “^T^”.

The genus *Moelleriella* encompasses two recognized, statistically robust clades: the Effuse Clade (BP = 94%, PP = 100%) and the Globose Clade (BP = 94%, PP = 100%) (Fig. 1). The Effuse clade was further divided into two sister subclades, designated as Subclade I and Subclade II. Subclade I encompassed three novel species identified in this study—*M.puwenensis*, *M.qionzhongensis*, and *M.multiperitheciata*—along with thirteen previously known species. Subclade II comprised four newly described species—*M.jinghongensis*, *M.hainanensis*, *M.pseudothanathonensis*, and *M.yuanyangensis*—as well as thirteen known species. The Globose clade included two new species—*M.yunnanensis* and *M.globostromata*—along with eight known species.

### ﻿Taxonomy

#### ﻿ Hypocrella limushanensis

Taxon classificationFungiHypocrealesClavicipitaceae

Hong Yu bis, Z.L. Yang, Z.Q. Wang & Jing Zhao sp. nov.

63A9093C-8EB9-595D-9316-32F9D9A9307E

851081

Fig. 2

##### Etymology.

Named after the Limu Mountains National Forest Park, where the species was collected.

##### Type.

China • Hainan Province, Qiongzhong County, the Limu Mountains National Forest Park, 19°23'N, 109°76'E, alt. 324 m, found on the underside of living leaves of dicotyledonous plants, 10 March 2023, Hong Yu (YHH 2303015, holotype; YFCC 23039299, ex-type).

**Figure 2.**
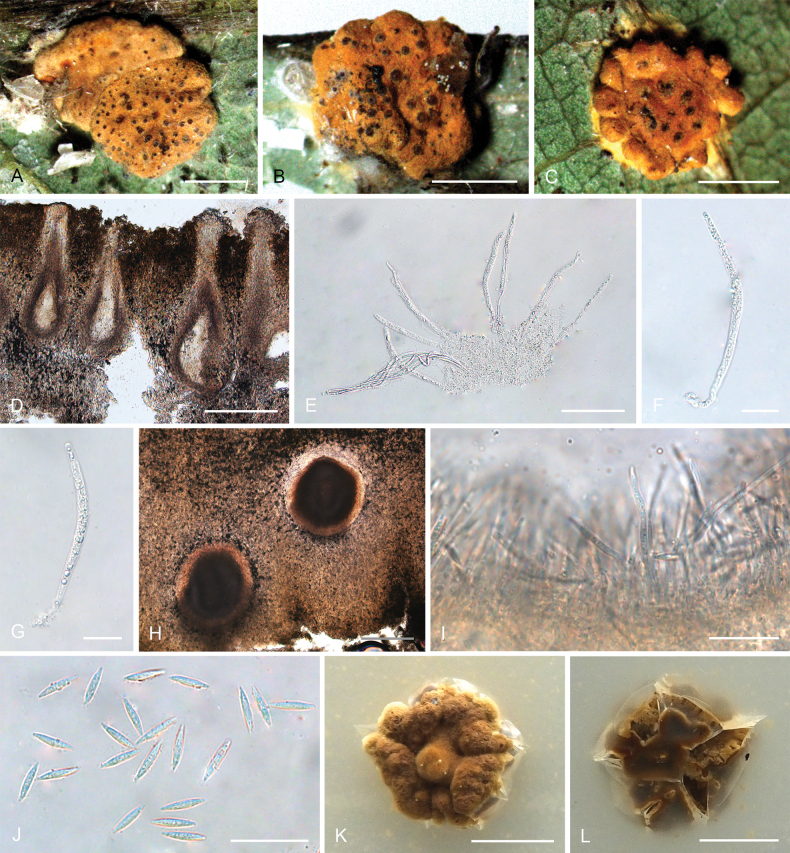
*Hypocrellalimushanensis***A, B** sexual stromata containing conidiomata and perithecia **C** asexual stroma containing conidiomata **D** perithecia **E–G** asci and ascospores **H, I** section of stromata showing conidiomata **J** conidia **K** colonies obverse on PDA at 25 °C after 21 days **L** colonies reverse on PDA at 25 °C after 21 days. Scale bars: 1 mm (**A–C**); 200 µm (**D**); 100 µm (**E**); 25 µm (**F–G**); 200 µm (**H**); 20 µm (**I–J**); 5 mm (**K–L**).

##### Description.

**Teleomorph: *Stromata*** pulvinate, brownish yellow, 1.9–3.5 mm, without hypothallus, surface smooth. ***Hyphae*** of stromata forming compact ***textura intricata*** to ***epidermoidea***. ***Perithecia*** embedded in stroma, numerous perithecia per stroma (>30), flask-shaped, 340–510 × 100–200 μm, ostioles projecting, dark brown to black. ***Asci*** mostly cylindrical, 85–135 × 5.0–7.5 μm, caps 1.4–3.0 μm thick. ***Ascospores*** hyaline, multi-septate, smooth, filiform to long fusiform. **Anamorph**: (Teleomorph not present with anamorph in the same stroma) ***Stromata*** generally pulvinate, yellow to dark orange, 1.8–2.0 mm in diameter with narrow hypothallus, surface smooth. ***Hyphae*** of stromata forming compact ***textura intricata*** to ***epidemoidea***. ***Conidiomata*** subglobose to globose, circularly arranged towards the center of stroma, a few conidiomata per stroma (≥5), 190–370 × 140–320 μm. ***Conidiomatal ostioles*** sunken, dark brown to dark. ***Phialides*** formed in a thick, compact palisade. ***Conidia*** hyaline, smooth, unicellular, fusoid with acute ends, 10–13 × 1.8–7.5 μm. ***Paraphyses*** present, linear, filiform, up to 42 µm long.

##### Culture characteristics.

Colonies on PDA slow-growing, attaining a diameter of 7–8 mm in 21 days at 25 °C. Stroma pulvinate, tomentose, surface wrinkled, brown to orange-brown, compact. Conidial masses typically do not develop within 21 days. Colony reverse brown to dark brown.

##### Habitat.

On scale insects (Coccidae, Hemiptera) or whiteflies (Aleyrodidae, Homoptera), found only on the underside of dicotyledonous leaves.

##### Distribution.

China, Hainan Province, Qiongzhong County.

##### Other materials examined.

China • Hainan Province, Qiongzhong County, the Limu Mountains National Forest Park, 19°23'N, 109°76'E, alt. 324 m, found on the underside of living leaves of dicotyledonous plants, 10 March 2023, Hong Yu (YHH 2303016, paratype; YFCC 23039301, ex-paratype); • Ibid., (YHH 2303017).

##### Notes.

Phylogenetic analyses indicated that *Hypocrellalimushanensis* constituted a sister lineage to H.cf.discoidea (strains I93-901D and I95-901D) and was grouped within the Pulvinate clade A (Fig. 1). This grouping was supported by the Bayesian Inference posterior probabilities (PP = 100%) and the maximum likelihood bootstrap proportions (BP = 100%). However, comprehensive morphological comparisons were not feasible due to the absence of documented morphological data for H.cf.discoidea.

#### ﻿ Hypocrella yunnanensis

Taxon classificationFungiHypocrealesClavicipitaceae

Hong Yu bis, Z.L. Yang, Z.Q. Wang & Jing Zhao sp. nov.

D173A914-8C04-52CA-95B3-F009A34ECD4E

851082

Fig. 3

##### Etymology.

Named after the location Yunnan Province, where this species was collected.

##### Diagnosis.

Similar to *H.discoidea* in having sunken conidiomata ostioles, *H.yunnanensis* differs by having narrow-oval conidia. It is similar to *H.limushanensis* in that it has subglobose to globose conidiomata and fusoid conidia, but it could be distinguished from *H.limushanensis* by smaller conidia.

**Figure 3.**
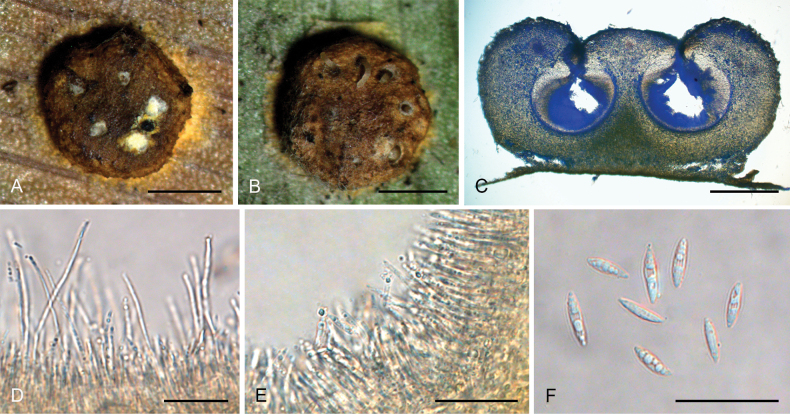
*Hypocrellayunnanensis***A, B** anamorphic stromata containing conidiomata **C** section of stroma showing conidiomata **D** phialides, conidia, and paraphyses **E** phialides with conidia (arrow) at the tips **F** conidia. Scale bars: 1 mm (**A, B**); 500 µm (**C**); 20 µm (**D–F**).

##### Type.

China • Yunnan Province, Jinping County, the Xi Long Mountains, 22°76'N, 102°55'E, alt. 1,715 m, found on the underside of living leaves of dicotyledonous plants, 28 May 2023, Hong Yu (YHH 2305020, holotype).

##### Description.

**Teleomorph**: Not known. **Anamorph: *Stromata*** generally discoid with slightly raised in the middle, brown to dark brown, 2.0–2.3 mm in diameter with narrow hypothallus, surface smooth. ***Stromata*** forming compact ***textura intricata*** to ***epidemoidea***. ***Conidiomata*** circularly arranged towards center of stroma with slightly dented, subglobose to globose, conidial masses white, greyish white, greyish to dark brown, a few conidiomata per stroma (≥5), 582–740 × 453–640 μm, with phialides formed in a thick compact palisade. ***Conidia*** unicellular, hyaline, smooth, fusoid with acute ends, 8.0–11 × 1.8–3.0 μm. ***Paraphyses*** present, linear, filiform, up to 92 µm long.

##### Habitat.

On scale insects (Coccidae, Hemiptera) or whiteflies (Aleyrodidae, Homoptera), found on the underside of dicotyledonous leaves.

##### Distribution.

China, Yunnan Province, Jinping County.

##### Other materials examined.

China • Yunnan Province, Jinping County, the Xi Long Mountains, 22°76'N, 102°55'E, alt. 1,715 m, found on the underside of living leaves of dicotyledonous plants, 28 May 2023, Hong Yu (YHH 2305021, paratype).

##### Notes.

Phylogenetic analyses demonstrated that *Hypocrellayunnanensis* constituted a distinct clade from other *Hypocrella* species, supported by high credibility values (PP = 100%, BP = 100%) (Fig. 1). This species exhibited a close relationship with *H.discoidea*, H.cf.discoidea, and *H.limushanensis*. Morphologically, *H.yunnanensis* shared similarities with *H.discoidea*, including sunken conidiomata ostioles, circularly arranged conidiomata towards the center of the stroma, and the presence of a hypothallus. However, key differences existed: *H.yunnanensis* features exhibited brown to dark brown stromata, subglobose to globose conidiomata, and narrow-oval conidia, whereas *H.discoidea* possessed red-brown stromata, flattened globose or laterally oval conidiomata, and fusoid conidia. Additionally, *H.yunnanensis* had shorter conidia (8.0–11 × 1.8–3.0 μm) and shorter paraphyses (up to 92 μm) compared to *H.discoidea* (10–15 × 1.5–2.0 μm and up to 180 μm, respectively) (Hywel-Jones and Evans 1993). Furthermore, *H.yunnanensis* resembled *H.limushanensis* in the formation of compact *textura intricata* to epidermoidea hyphae, subglobose to globose conidiomata arranged circularly towards the center of the stroma, sunken conidiomatal ostioles, and fusoid conidia. Nevertheless, it could be differentiated from *H.limushanensis* by the color of the stromata (brown to dark brown vs. yellow to dark orange), smaller conidia (8.0–11 × 1.8–3.0 μm vs. 10–13 × 1.8–7.5 μm), larger conidiomata (582–740 × 453–640 μm vs. 190–370 × 140–320 μm), and longer paraphyses (up to 92 μm vs. up to 42 μm).

#### ﻿ Moelleriella globostromata

Taxon classificationFungiHypocrealesClavicipitaceae

Hong Yu bis, Z.L. Yang & Z.Q. Wang sp. nov.

2A8FC3A3-C48F-50D8-9438-522F9C74B271

851083

Fig. 4

##### Etymology.

*globostromata*, referring to the macro-morphological characteristics of the stromata like a sphere.

##### Diagnosis.

Similar to *Moelleriellaboliviensis* by having completely embedded perithecia, but *M.globostromata* can be clearly distinguished by bigger stromata and smaller perithecia.

##### Type.

China • Yunnan Province, Jinghong City, Jinuo Village, 22°06'N, 100°98'E, alt. 1,035 m, found on the underside of living leaves of dicotyledonous plants, 2 October 2022, Hong Yu (YHH 221009, holotype; YFCC 22109275, ex-type).

**Figure 4.**
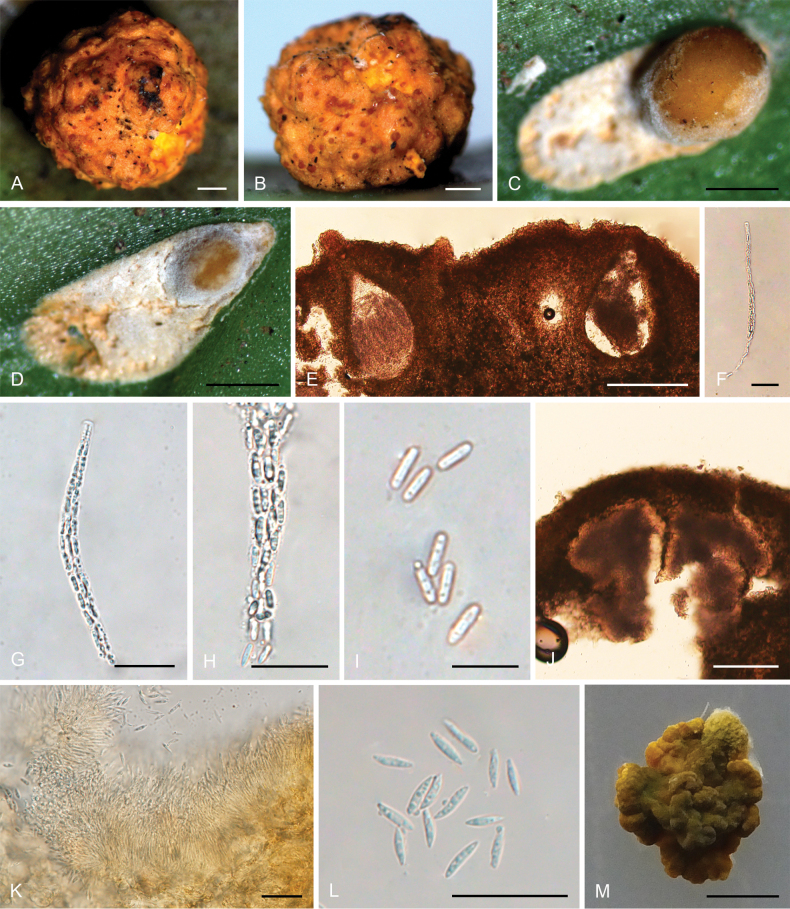
*Moelleriellaglobostromata***A, B** teleomorphic stromata containing perithecia **C, D** anamorphic stromata containing conidiomata **E** perithecia **F–H** asci and part-spores **I** part-spores **J, K** section of stroma showing conidiomata **L** conidia **M** colonies obverse on PDA at 25 °C after 21 days. Scale bars: 1 mm (**A–D**); 200 µm (**E**); 25 µm (**F**); 20 µm (**G–H**); 10 µm (**I**); 100 µm (**J**); 20 µm (**K**); 20 µm (**L**); 5 mm (**M**).

##### Description.

**Teleomorph: *Stromata*** pulvinate and slightly tuberculate, globose, slightly constricted at base, 5.0–7.0 mm in diameter, pale orange, without hypothallus. ***Hyphae*** of stromata forming compact ***textura epidermoidea***. ***Perithecia*** densely arranged in the stromata, completely embedded, stroma slightly raised where the perithecia are present, numerous perithecia per stroma (>90), ostioles projecting, brownish-yellow. ***Perithecia*** flask-shaped, 110–410 × 110–205 μm. ***Asci*** cylindrical, 85–170 × 3.0–6.3 μm, caps 0.8–1.8 μm thick. ***Ascospores*** initially filiform, disarticulating into part-spores. ***Part-spores*** short-cylindrical with rounded or blunt ends, 4.0–7.0 × 1.2–2.5 μm. **Anamorph**: (The teleomorph and anamorph are not found in the same stroma.) ***Stromata*** thin pulvinate with subglobose to globose tuberculate at one end of stroma, tomentose, white, orange-pink to reddish brown, 3.7–4.1 × 1–1.5 mm. In section, conidioma U-shaped. ***Conidia*** unicellular, hyaline, smooth, fusiform with acute ends, 6.0–10 × 1.2–2.0 μm. No paraphyses observed.

##### Culture characteristics.

Colonies on PDA slow-growing, attaining a diameter of 8.0–9.0 mm in 21 days at 25 °C. Stromatic colonies compact pulvinate, surface wrinkled, pale orange, yellowish brown to greyish green. The conidial mass was not produced in 21 days.

##### Habitat.

On scale insects (Coccidae, Hemiptera) or whiteflies (Aleyrodidae, Homoptera), found on the underside of dicotyledonous leaves.

##### Distribution.

China, Yunnan Province, Jinghong City, and Pu’er City.

##### Other materials examined.

China • Yunnan Province, Jinghong City, Jinuo Village, 22°06'N, 100°98'E, alt. 1,035 m, found on the underside of living leaves of dicotyledonous plants, 2 October 2022, Hong Yu (YHH 221010, paratype; YFCC 22109276, ex-paratype); Ibid., (YHH 221011). China • Yunnan Province, Pu’er City, Nandaohe Village, Yeyatang, 22°60'N, 100°99'E, alt. 1,000 m, found on the underside of living leaves of dicotyledonous plants, 3 August 2023, Hong Yu (YHH 2308021, YHH 2308022, YHH 2308023). China • Yunnan Province, Jinghong City, Jinuo Village; 22°06'N, 100°98'E, alt. 1,046 m, found on the underside of living leaves of monocotyledonous plants (banana tree), 26 September 2023, Hong Yu (YHH 2309007, YHH 2309008).

##### Notes.

Phylogenetic analyses revealed that *Moelleriellaglobostromata* constituted a distinct clade within the Globose clade, supported by the Bayesian Inference posterior probabilities (PP = 83%) and the maximum likelihood bootstrap proportions (BP = 83%) (Fig. 1). This species was closely related to *M.insperata* and *M.boliviensis*. In the present study, both the teleomorph and anamorph of stromata were observed in *M.globostromata*. However, for *M.insperata*, only the anamorph of stromata had been documented, and for *M.boliviensis*, only the teleomorph of stromata had been reported (Liu and Hodge 2005; Chaverri et al. 2008).

#### ﻿ Moelleriella hainanensis

Taxon classificationFungiHypocrealesClavicipitaceae

Hong Yu bis, Z.L. Yang, Z.Q. Wang & Jing Zhao sp. nov.

06A94557-EAA2-5790-9DB5-A070E00AF10E

851089

Fig. 5

##### Etymology.

Named after the location Hainan Province, where this species was collected.

##### Diagnosis.

Similar to *Moelleriellasimaoensis* by producing tubercles in stromata, but *M.hainanensis* differed from *M.simaoensis* by having no anamorph distribution in the center.

**Figure 5.**
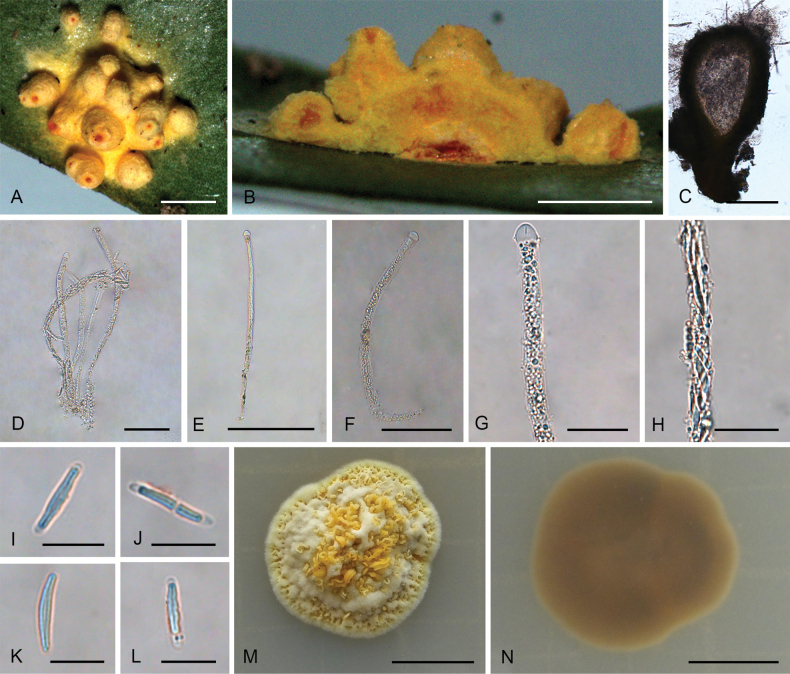
*Moelleriellahainanensis***A, B** teleomorphic stroma containing perithecia **C** perithecia **D–H** asci and part-spores **I–L** part-spores **M** colonies obverse on PDA at 25 °C after 21 days **N** colonies reverse on PDA at 25 °C after 21 days. Scale bars: 1 mm (**A, B**); 200 µm (**C**); 50 µm (**D**); 100 µm (**E**); 50 µm (**F**); 20 µm (**G–H**); 10 µm (**I–L**); 1 cm (**M–N**).

##### Type.

China • Hainan Province, Hainan Province, Qiongzhong County, the Limu Mountains National Forest Park, 19°23'N, 109°76'E, alt. 324 m, found on the underside of living leaves of dicotyledonous plants, 10 March 2023, Hong Yu (YHH 2303020, holotype; YFCC 23039277, ex-type).

##### Description.

**Teleomorph: *Stromata*** effuse to thin pulvinate with pronounced gregarious tubercles, yellow, 3.0–4.0 mm in diameter, 1.1–1.3 mm in height, surface tomentose, opaque, with hyaline to yellow thin hypothallus. ***Hyphae*** of stromata forming loose ***textura intricata*** to ***epidermoidea***. Tubercles strongly projecting and aggregated, ovoid or subglobose, ostioles reddish orange, numerous perithecia per stroma (>10). ***Perithecia*** embedded in tubercles, one perithecium per tubercle, perithecia narrowly ovoid, 210–460 × 150–310 μm. ***Asci*** cylindrical, 120–270 × 4.2–10 μm, caps 3.4–6.3 μm thick. ***Ascospores*** filiform, multi-septate, disarticulating into part-spores that cylindrical to fusoid with rounded ends, 10–17 × 1.5–3.5 μm. **Anamorph**: Not known.

##### Culture characteristics.

Colonies on PDA moderate-growing, attaining a diameter of 21–22 mm in 21 days at 25 °C. Stromatic colonies white, pale yellow to yellow, compact, forming a pulvinate structure. Conidial masses usually abundant, pale yellow to yellow. Colony reverse side yellowish brown, white to pale yellow at the margins.

##### Habitats.

On scale insects (Coccidae, Hemiptera) or whiteflies (Aleyrodidae, Homoptera), found on the underside of dicotyledonous leaves.

##### Distribution.

China, Hainan Province, Qiongzhong County.

##### Notes.

Phylogenetic analyses revealed that *Moelleriellahainanensis* formed a sister lineage with *M.simaoensis* and was closely related to *M.puerensis*, *M.raciborskii*, *M.pseudothanathonensis*, and *M.thanathonensis* (Fig. 1). However, only the anamorph morphology of *M.thanathonensis* was reported, while only the anamorph stromata of species *M.pseudothanathonensis* were collected in this study, so *M.hainanensis* could not be compared with *M.pseudothanathonensis* and *M.thanathonensis* in detail. *Moelleriellahainanensis* was similar to *M.simaoensis*, *M.puerensis*, and *M.raciborskii* by producing tubercles in stromata (Liu et al. 2006; Wang et al. 2022; Zhang et al. 2023). However, it differed from *M.simaoensis*, *M.puerensis*, and *M.raciborskii* in that there was no anamorph distribution in the center, while the tubercles of these three species were located at the margin and the anamorph was in the center (Liu et al. 2006; Wang et al. 2022; Zhang et al. 2023).

#### ﻿ Moelleriella jinghongensis

Taxon classificationFungiHypocrealesClavicipitaceae

Hong Yu bis, Z.L. Yang, Z.Q. Wang & J.M. Ma sp. nov.

93E82A44-C532-5CFD-B41A-07C133B368EE

851090

Fig. 6

##### Etymology.

Named after the location Jinghong City, where the species was collected.

##### Type.

China • Yunnan Province, Jinghong City, Puwen Town, 22°52'N, 100°97'E, alt. 1,020 m, found on the underside of living leaves of dicotyledonous plants, 3 August 2023, Hong Yu (YHH 2308025, holotype; YFCC 23089312, ex-type).

**Figure 6.**
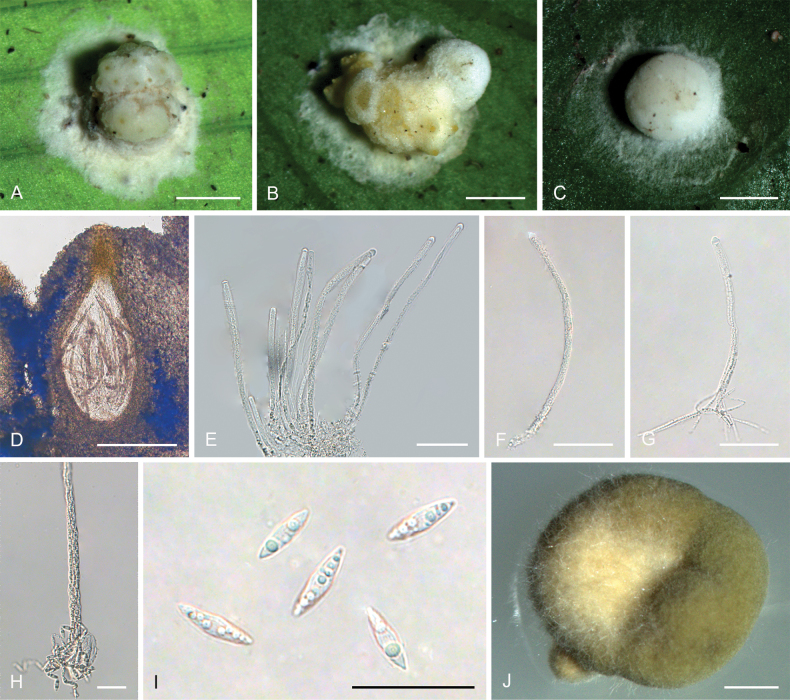
*Moelleriellajinghongensis***A, B** teleomorphic stroma containing perithecia **C** immature stromata **D** perithecia **E–G** mature asci with developing asci **H** asci and part-spores **I** conidia **J** colonies obverse on PDA at 25 °C after 21 days. Scale bars: 1 mm (**A–C**); 200 µm (**D**); 50 µm (**E–G**); 25 µm (**H**); 20 µm (**I**); 1 mm (**J**).

##### Description.

**Teleomorph: *Stromata*** white, greyish white to yellowish white, pulvinate and slightly tuberculate, subglobose to globose, surface tomentose, 2.5–3.0 mm in diameter, slightly constricted or constricted at base, surrounded by thin hypothallus. Stromatal tissue dense ***textura intricata***. ***Perithecia*** densely arranged in the stroma, completely embedded, obpyriform, numerous perithecia per mature stroma (>15), 278–568 × 170–215 μm, ostioles usually not projecting and sometimes projecting, yellow to brown yellow. ***Asci*** cylindrical, 165–240 × 6.0–9.0 μm, asci caps umbonate, 3.5–5.2 μm thick. ***Ascospores*** initially filiform, dividing into part-spores that fusoid, acute at both ends, 10–14.5 × 2.5–3.7 μm. **Anamorph**: Not known.

##### Culture characteristics.

Colonies on PDA slow-growing, attaining a diameter of 4.0–6.0 mm in 21 days at 25 °C. Stromatic colonies pale yellow, surface tomentose, slight depression in the center. No conidial masses were observed.

##### Habitat.

On scale insects (Coccidae, Hemiptera) or whiteflies (Aleyrodidae, Homoptera), found on the underside of living leaves of dicotyledonous plants.

##### Distribution.

China, Yunnan Province, Jinghong City.

##### Other materials examined.

China • Yunnan Province, Jinghong City, Puwen Town, 22°52'N, 100°97'E, alt. 1,020 m, found on the underside of living leaves of dicotyledonous plants, 3 August 2023, Hong Yu (YHH 2308026, paratype; YFCC 23089313, ex-paratype); • Ibid., (YHH 2308027, YHH 2308028).

##### Notes.

Phylogenetic analyses revealed that *Moelleriellajinghongensis* was closely related to *M.rhombispora* and *M.yuanyangensis*, with the latter being the sister species to *M.jinghongensis* (Fig. 1). However, in this study, only the teleomorphic stromata of *M.jinghongensis* and the anamorphic stromata of *M.yuanyangensis* were collected, precluding a detailed morphological comparison between the two species. *Moelleriellajinghongensis* exhibited similarities to *M.rhombispora* in terms of its dense stromatal tissue, embedded and densely arranged perithecia, cylindrical asci, and fusoid part-spores. Nevertheless, *M.jinghongensis* could be distinguished by its distinct stromatal color and size, narrower perithecia, and shorter asci-cap (Chaverri et al. 2008).

#### ﻿ Moelleriella multiperitheciata

Taxon classificationFungiHypocrealesClavicipitaceae

Hong Yu bis, Z.L. Yang, Z.Q. Wang & J.M. Ma sp. nov.

7CBA8D89-B930-510A-8AE6-BFC85869F832

851091

Fig. 7

##### Etymology.

*multiperitheciata*, indicating that the number of perithecia was very much from the mature specimens; “*multi*” means plenty of perithecia.

**Figure 7.**
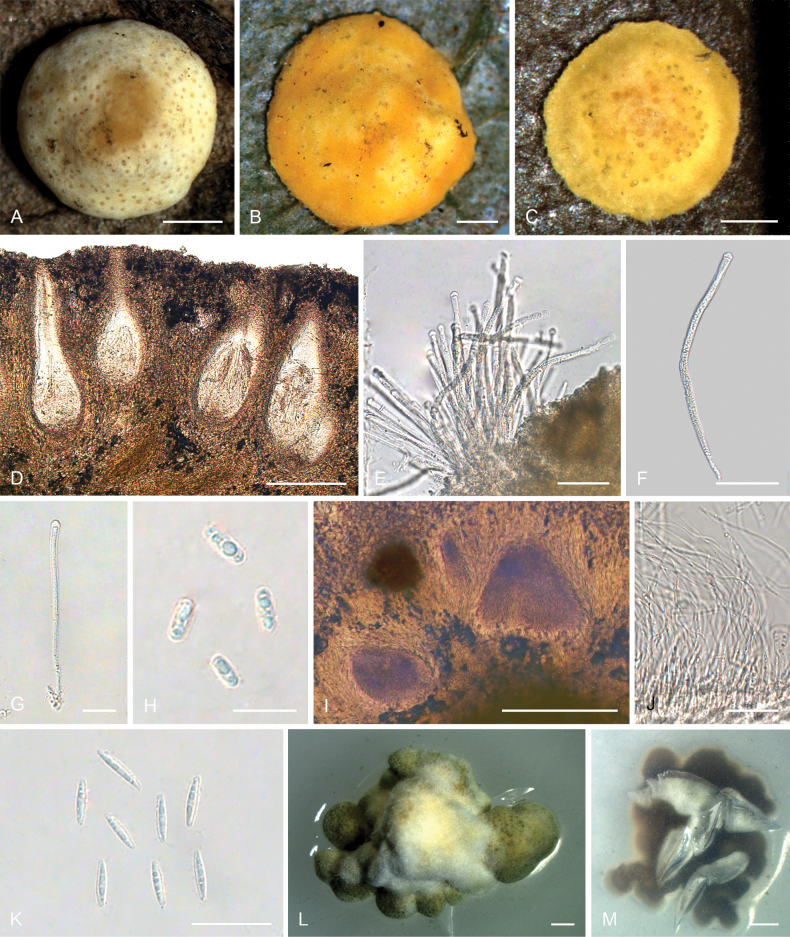
*Moelleriellamultiperitheciata***A, B** teleomorphic stroma containing perithecia **C** anamorphic stroma containing conidiomata **D** perithecia **E–G** mature asci with developing asci **H** part-spores **I** Section of stroma showing conidiomata **J** paraphyses **K** conidia **L** colonies obverse on PDA at 25 °C after 21 days **M** colonies reverse on PDA at 25 °C after 21 days. Scale bars: 1 mm (**A, B**); 500 µm (**C**); 200 µm (**D**); 50 µm (**E–F**); 25 µm (**G**); 10 µm (**H**); 200 µm (**I**); 20 µm (**J–K**); 1 mm (**L–M**).

##### Diagnosis.

Similar to *Moelleriellaphukhiaoensis* in having flask-shaped perithecia and cylindrical part-spores, *M.multiperitheciata* could be distinguished by its smaller perithecia, smaller asci, and smaller conidia.

##### Type.

China • Yunnan Province, Pu’er City, Nandaohe Village, Yeyatang, 22°60'N, 100°99'E, alt. 1,000 m, found on the leaves that have fallen to the ground and the underside of living leaves of dicotyledonous plants, 3 August 2023, Hong Yu (YHH 2308010, holotype; YFCC 23089307, ex-type).

##### Description.

**Teleomorph: *Stromata*** pulvinate, mature stromata have a larger bulgeis, immature stromata slightly convex, hemi-globose with a hemispheric central region abruptly attenuating and towards the edge, or scutate, yellow white when mature, yellow to orange yellow when immature, 3.3–3.8 mm in diameter, surface smooth, without hypothallus. ***Perithecia*** densely arranged in stromata, numerous perithecia per mature stroma (>220), fully embedded, flask-shaped, 285–435 × 90–155 μm, ostioles not projecting, yellowish brown. ***Asci*** cylindrical, 90–145 × 3.0–6.0 μm, caps 3.5–5.0 μm thick. ***Ascospores*** initially filiform, dividing into part-spores. ***Part-spores*** cylindrical with rounded ends, 4.5–11 × 1.8–2.8 μm. **Anamorph**: (The teleomorph and anamorph are not found in the same stroma.) ***Stromata*** on natural substrate usually pulvinate, surface tomentose, yellow, 1.1–1.8 mm in diameter. ***Conidiomata*** scattered in stromata, numerous conidiomata per stroma (>60). In section, the conidioma subglobose to globose, 86–206 × 38–97 μm. ***Conidial masses*** orange yellow in stromata central, with a yellow-green in the periphery. With phialides formed in a thick compact palisade, phialides cylindrical, 5.0–13 μm long. ***Conidia*** unicellular, hyaline, smooth, fusiform with acute ends, 10–13.6 × 1.6–3.0 μm. ***Paraphyses*** present, linear, filiform, up to 160 µm long.

##### Culture characteristics.

Colonies on PDA slow-growing, attaining a diameter of 10–13 mm in 21 days at 25 °C. Stromatic colonies white, pale yellow to greyish green, surface tomentose. Colony reverse side sepia and pale orange at the margin.

##### Habitat.

On scale insects (Coccidae, Hemiptera) and whiteflies (Aleyrodidae, Homoptera), found on leaves that have fallen to the ground and the underside of living leaves of dicotyledonous plants.

##### Distribution.

China, Yunnan Province, Pu’er City.

##### Other materials examined.

China • Yunnan Province, Pu’er City, Nandaohe Village, Yeyatang, 22°60'N, 100°99'E, alt. 1,000 m, found on the leaves that have fallen to the ground and the underside of living leaves of dicotyledonous plants, 3 August 2023, Hong Yu (YHH 2308011, paratype; YFCC 22109308, ex-paratype); • Ibid., (YHH 2308012, YHH 2308013, YHH 2308014, YHH 230815, YHH 2308016; YFCC 22109309, living culture). China • Yunnan Province, Pu’er City, Simao District, Meizihu Park, 22°71'N, 100°96'E, alt. 1,329 m, 3 August 2023, Hong Yu (YHH 2308032). China • Yunnan Province, Pu’er City, Simao District, Xinfang Reservoir, 22°71’ N, 100°95’ E, alt. 1,329 m, 2 August 2023, Hong Yu (YHH 2308033, YHH 2308034).

##### Notes.

Phylogenetic analyses revealed that *Moelleriellamultiperitheciata* was clustered within the Effuse clade and was a sister species to *M.phukhiaoensis* (Fig. 1). Both species exhibited embedded, flask-shaped perithecia, cylindrical part-spores, and paraphyses. However, *M.multiperitheciata* could be differentiated from *M.phukhiaoensis* by its yellowish-brown coloration, non-projecting ostioles, fewer perithecia per mature stroma, smaller perithecia (285–435 × 90–155 μm vs. 400–520 × 150–200 μm), smaller asci (90–145 × 3.0–6.0 μm vs. 195–220 × 8.0–12 μm), smaller conidiomata (86–206 × 38–97 μm vs. 430 × 100 μm), smaller conidia (10–13.6 × 1.6–3.0 μm vs. 16–17 × 2.5–3.5 μm), and longer paraphyses (up to 160 μm vs. up to 90 μm) (Li et al. 2016). Ecologically, *M.multiperitheciata* was frequently observed on fallen leaves and the undersides of living leaves of dicotyledonous plants, whereas *M.phukhiaoensis* was typically found on the undersides of living leaves of dicotyledonous plants.

#### ﻿ Moelleriella pseudothanathonensis

Taxon classificationFungiHypocrealesClavicipitaceae

Hong Yu bis, Z.L. Yang & Z.Q. Wang sp. nov.

652480AB-8CBF-54E3-B23E-6102DC5EF4B9

851092

Fig. 8

##### Etymology.

Referring to morphologically resembling *Moelleriellathanathonensis* but phylogenetically distinct.

**Figure 8.**
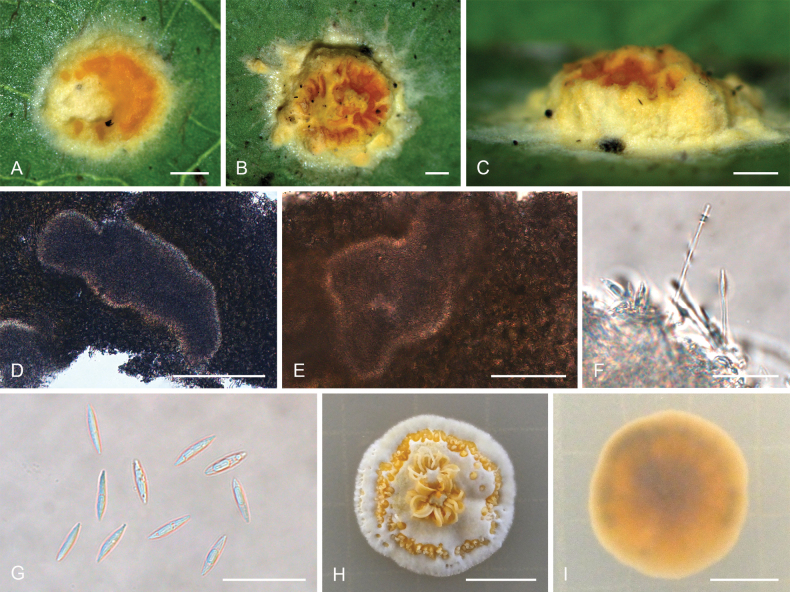
*Moelleriellapseudothanathonensis***A–C** anamorphic stromata containing conidiomata **D, E** section of stromata showing conidiomata **F** phialides and paraphyses **G** conidia **H** colonies obverse on PDA at 25 °C after 21 days **I** colonies reverse on PDA at 25 °C after 21 days. Scale bars: 1 mm (**A–C**); 200 µm (**D**); 100 µm (**E**); 20 µm (**F–G**); 1 cm (**H–I**).

##### Diagnosis.

Similar to *M.puerensis* in producing fusoid conidia, but it differed from *M.puerensis* by its stromata being concave on one side and without conidiomata on the concavity.

##### Type.

China • Yunnan Province, Yuanyang County, Xinjie Town, 23°08'N, 102°86'E, alt. 2,054 m, found on the underside of living leaves of dicotyledonous plants, 25 September 2022, Hong Yu (YHH 2209004, holotype; YFCC 22099302, ex-type).

##### Description.

**Teleomorph**: Not known. **Anamorph: *Stromata*** thin pulvinate when immature, sometimes almost cylindrical when mature, one side concave and without conidiomata on the concavity, cream-colored to yellow, 4.4–4.7 mm, surrounded by narrow hypothallus. ***Hyphae*** of stromata forming loose ***textura intricata*** to ***epidemoidea***. ***Conidiomata*** solitary or gregarious, simple depressions of surface without distinct rims, irregular in shape, multi-locular, several conidiomata per stroma, but difficult to count because they fuse with neighboring ones, widely open. ***Paraphyses*** present. ***Conidial masses*** orange yellow, orange to deep orange. In section, conidioma irregular-shaped. With phialides formed in a thick compact palisade, phialides cylindrical. ***Conidia*** unicellular, hyaline, smooth, fusoid with acute ends, 10–12.5 × 1.6–2.7 μm.

##### Culture characteristics.

Colonies on PDA moderate-growing, attaining a diameter of 20–21 mm in 21 days at 25 °C. Stromatic colonies pulvinate, tomentose, white. Conidial masses in the center forming several gushing bands and others in a ring towards the center of colonies distribution, abundant, confluent, cream-colored to yellow. Colony reverse side pale yellow, yellow, dark brown to black.

##### Habitat.

On scale insects (Coccidae, Hemiptera) and whiteflies (Aleyrodidae, Homoptera), found on the underside of dicotyledonous leaves.

##### Distribution.

China, Yunnan Province, Yuanyang County.

##### Other materials examined.

China • Yunnan Province, Yuanyang County, Xinjie Town, 23°08'N, 102°86'E, alt. 2,054 m, found on the underside of living leaves of dicotyledonous plants, 25 September 2022, Hong Yu (YHH 2209005, paratype; YFCC 22099303, ex-paratype).

##### Notes.

Phylogenetic analyses revealed that *Moelleriellapseudothanathonensis* constituted a distinct clade, positioned as a sister taxon to *M.puerensis* (Fig. 1). This species exhibited similarities with *M.puerensis* in terms of producing conidiomata characterized by simple surface depressions devoid of distinct margins, wide openings, fusiform conidia, and the presence of paraphyses (Wang et al. 2022). Nevertheless, *M.pseudothanathonensis* could be differentiated from *M.puerensis* by its stromata, which were concave on one side and lacked conidiomata in the concave region. Furthermore, *M.pseudothanathonensis* shared similar morphological features with *M.thanathonensis* regarding stromata structure. However, it was distinguishable from *M.thanathonensis* by the concavity on one side of the stromata, the absence of conidiomata in this area, and the formation of multiple eruptive bands of fungal colonies within the central conidial mass when cultured on PDA.

#### ﻿ Moelleriella puwenensis

Taxon classificationFungiHypocrealesClavicipitaceae

Hong Yu bis, Z.L. Yang, Z.Q. Wang & J.M. Ma sp. nov.

1A4B7631-E92E-508F-B977-DF6AE4BE895B

851093

Fig. 9

##### Etymology.

Named after the location Puwen Town, where the species was collected.

**Figure 9.**
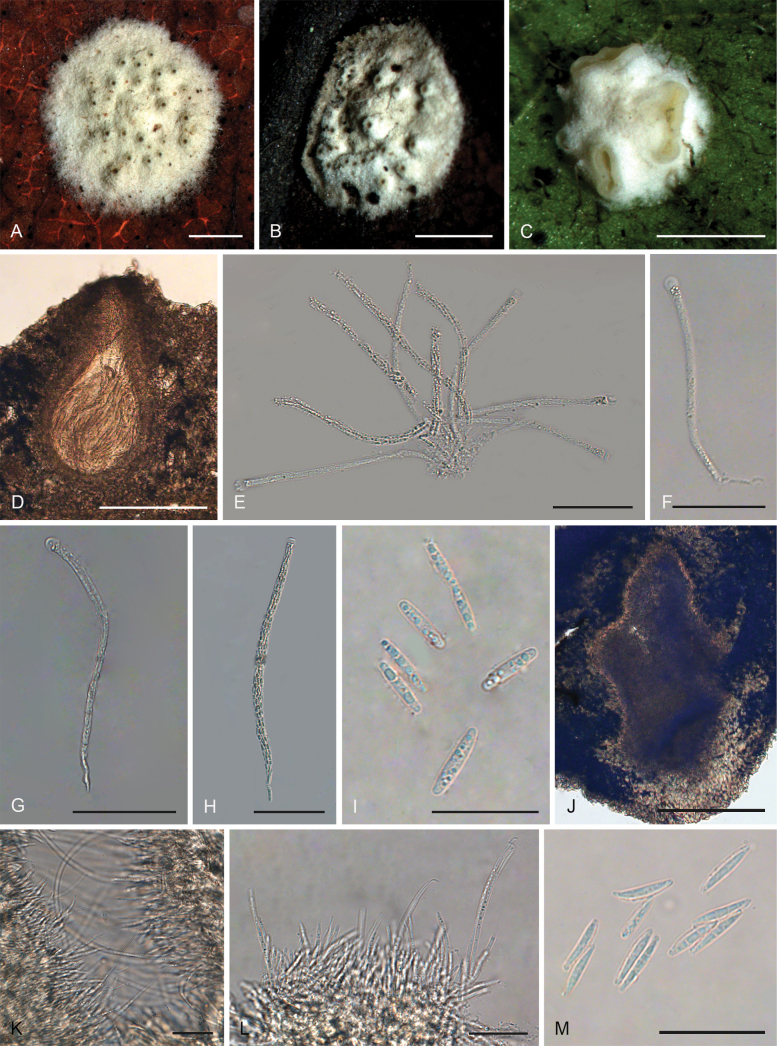
*Moelleriellapuwenensis***A, B** teleomorphic stromata containing perithecia **C** anamorphic stroma containing conidiomata **D** perithecia **E–H** mature asci with developing asci **I** part-spores **J** section of stroma showing conidiomata **K–L** phialides and paraphyses (arrow) **M** conidia. Scale bars: 1 mm (**A–C**); 100 µm (**D**); 50 µm (**E–H**); 20 µm (**I**); 200 µm (**J**); 10 µm (**K–M**).

##### Diagnosis.

Similar to *Moelleriellachiangmaiensis* in having a widely open orifice and a thick and raised rim around the orifice, it could be distinguished from *M.chiangmaiensis* by its smaller perithecia and longer conidia.

##### Type.

China • Yunnan, Jinghong City, Puwen Town. Collections were from the rotting leaves and the underside of living leaves of dicotyledonous plants, 22°52'N, 100°97'E, alt. 1,020 m, 3 August 2023, Hong Yu (YHH 2308029, holotype).

##### Description.

**Teleomorph: *Stromata*** flattened pulvinate with subglobose tubercles, some tubercles fused together, but more often discrete, surface tomentose, white to moderate yellow, 2.0–3.5 mm, with globose to subglobose base, without hypothallus. ***Perithecia*** develop singly in tubercles, semi-embedded, obpyriform, 309–440 × 125–236 μm, numerous perithecia per stroma (>25). ***Ostioles*** not projecting, dark brown to black. ***Asci*** cylindrical, 95–190 × 3.3–5.9 μm, caps 3.0–7.7 μm thick. ***Ascospores*** initially filiform, disarticulating into part-spores. ***Part-spores*** cylindrical with rounded ends, 8.0–14.5 × 1.6–2.7 μm. **Anamorph**: (The teleomorph and anamorph are not found in the same stroma.) ***Stromata*** on natural substrate usually pulvinate, surface tomentose, white to moderate yellow, 1.3–1.5 mm in diameter. ***Conidiomata*** scattered in stromata, widely open orifice, rim around the orifice thick and raised, resembles an elongated lip, 350–545 × 185–250 μm, a few perithecia per stroma. Paraphyses present, linear, filiform, up to 85 µm long. ***Conidial masses*** cream-colored. With phialides formed in a thick compact palisade, phialides cylindrical, unicellular, 8.4–18 × 0.7–1.7 μm. ***Conidia*** unicellular, hyaline, smooth, fusiform with acute ends, 10–15 × 1.2–1.9 μm.

##### Habitat.

On scale insects (Coccidae, Hemiptera) and whiteflies (Aleyrodidae, Homoptera), found on leaves that have fallen to the ground and the underside of living leaves of dicotyledonous plants.

##### Distribution.

China, Yunnan Province, Jinghong City.

##### Other materials examined.

China • Yunnan, Jinghong City, Puwen Town. Collections were from the rotting leaves and the underside of living leaves of dicotyledonous plants, 22°52'N, 100°97'E, alt. 1,020 m, 3 August 2023, Hong Yu (YHH 2308030, YHH 2308031, paratype).

##### Notes.

*Moelleriellapuwenensis* was similar to the phylogenetic sister species *M.chiangmaiensis* (Fig. 1) in having thin, white to moderate yellow stromata, semi-embedded and obpyriform perithecia, part-spores cylindrical with rounded ends, and scattered conidiomata, the widely open orifice, and a thick and raised rim around the orifice. However, it could be distinguished from *M.chiangmaiensis* by its smaller stroma, smaller perithecia (309–440 × 125–236 vs. 450–500 × 260–360), and bigger part-spores and longer conidia (10–15 × 1.2–1.9 vs. 8.0–10 × 1.5–2.0) (Khonsanit et al. 2021).

#### ﻿ Moelleriella qionzhongensis

Taxon classificationFungiHypocrealesClavicipitaceae

Hong Yu bis, Z.L. Yang, Z.Q. Wang & Jing Zhao sp. nov.

04540144-4160-516B-8F77-4CC79DC05B41

851094

Fig. 10

##### Etymology.

Named after the location Qionzhong County, where this species was collected.

**Figure 10.**
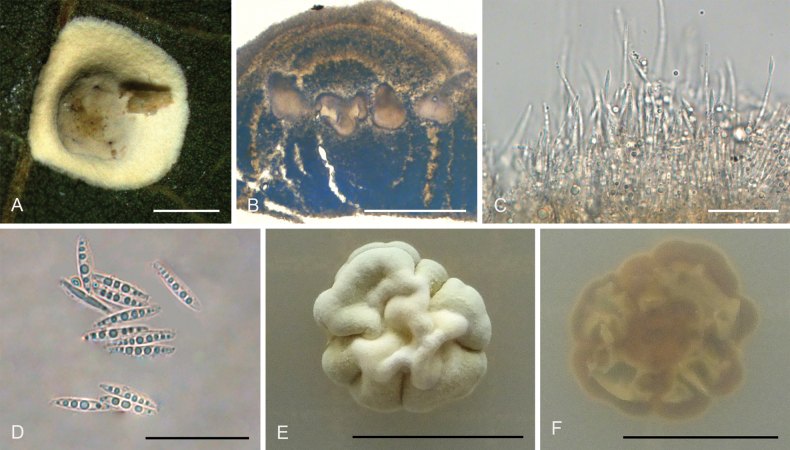
*Moelleriellaqionzhongensis***A** anamorphic stroma containing conidiomata **B** section of stroma showing conidiomata **C** phialides and paraphyses (arrow) **D** conidia **E** colonies obverse on PDA at 25 °C after 21 days **F** colonies reverse on PDA at 25 °C after 21 days. Scale bars: 1 mm (**A**); 500 µm (**B**); 20 µm (**C**); 20 µm (**D**); 1 cm (**E–F**).

##### Diagnosis.

It was similar to *Moelleriellaflava* in the shape of an anamorphic stroma, but it differed from *M.flava* by the color of the stromata and longer paraphyses.

##### Type.

China • Hainan Province, Qiongzhong County, the Limu Mountains National Forest Park, 19°23'N, 109°76'E, alt. 324 m, found on the underside of living leaves of dicotyledonous plants, 10 March 2023, Hong Yu (YHH 2303021, holotype; YFCC 23039306, ex-type).

##### Description.

**Teleomorph**: Not known. **Anamorph: *Stromata*** thin pulvinate with subglobose umbonate in the center, 2.4–2.6 mm in diameter, white to brownish grey, surface tomentose, without hypothallus. ***Hyphae*** of stromata forming loose ***textura intricata*** to ***epidemoidea***. ***Conidioma*** is situated in the center of stroma, with few conidioma per stroma. In section, the conidioma irregular-shaped, small, neatly arranged in a row. With phialides formed in a thick compact palisade, phialides cylindrical, 16–22 μm long. ***Conidia*** unicellular, hyaline, smooth, fusoid with acute ends, 10–15 × 1.4–3.3 μm. ***Paraphyses*** present, linear, filiform, up to 65 µm long.

##### Culture characteristics.

Colonies on PDA slow-growing, attaining a diameter of 8.0–9.0 mm in 21 days at 25 °C. Stromatic colony pulvinate, surface wrinkled and tomentose, white to pale greyish white. Colonies reverse side white, yellow to yellowish brown.

##### Habitat.

On scale insects (Coccidae, Hemiptera) and whiteflies (Aleyrodidae, Homoptera), found only on the underside of dicotyledonous leaves.

##### Distribution.

China, Hainan Province, Qiongzhong County.

##### Other materials examined.

China • Hainan Province, Qiongzhong County, the Limu Mountains National Forest Park, 19°23'N, 109°76'E, alt. 324 m, found on the underside of living leaves of dicotyledonous plants, 10 March 2023, Hong Yu (YHH 2303022, YHH 2303023, paratype).

##### Notes.

The multi-locus phylogenetic analysis demonstrated that the two samples of *Moelleriellaqionzhongensis* clustered together with strong statistical support (PP = 100%; BP = 100%), and they formed a sister clade to *M.flava* (Fig. 1). Morphologically, *M.qionzhongensis* exhibited similarities to *M.flava* in the structure of the anamorphic stroma (thin pulvinate with a subglobose umbonate center). However, *M.qionzhongensis* could be distinguished from *M.flava* based on the color of the stromata, the shape of the conidioma (irregular-shaped versus narrowly U-shaped), and the presence of longer paraphyses. Additionally, the conidia of *M.flava* possessed a thickened lateral wall, which was absent in *M.qionzhongensis*.

#### ﻿ Moelleriella yuanyangensis

Taxon classificationFungiHypocrealesClavicipitaceae

Hong Yu bis, Z.L. Yang & Z.Q. Wang sp. nov.

99113A67-7CB6-56ED-B940-3B0A18A05D82

851095

Fig. 11

##### Etymology.

Named after the location of the holotype collection—Yuanyang County, which is famous for its terraced landscape.

**Figure 11.**
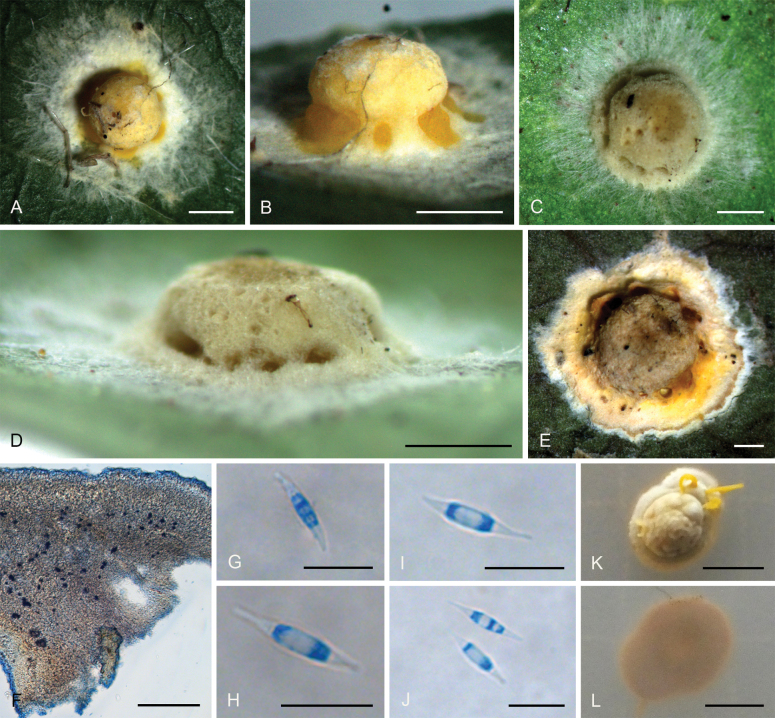
*Moelleriellayuanyangensis***A–E** anamorphic stromata containing conidiomata **F** section of stroma showing conidiomata **G–J** conidia **K** colonies obverse on PDA at 25 °C after 21 days **L** colonies reverse on PDA at 25 °C after 21 days. Scale bars: 1 mm (**A–E**); 200 µm (**F**); 10 µm (**G–J**); 5 mm (**K–L**).

##### Diagnosis.

It could be distinguished from other species of *Moelleriella* by its stromatal morphology and ventricose conidia.

##### Type.

China • Yunnan Province, Yuanyang County, Xinjie Town, 23°08'N, 102°86'E, alt. 2,054 m, found on the underside of living leaves of dicotyledonous plants, 25 September 2022, Hong Yu (YHH 2209001, holotype; YFCC 23039314, ex-type).

##### Description.

**Teleomorph**: Not known. **Anamorph: *Stromata*** with a globose head, gradually expanding towards the base, base wide, greyish yellow when immature, becoming yellow, deep yellow, orange to greyish-brown when mature, 1.4–3.5 mm in diameter, surface tomentose and base pruinose due to loosely woven, with hypothallus in immature stromata, 0.6–2.2 mm wide. ***Hyphae*** of stromata forming loose ***textura intricata*** to ***epidermoidea***. ***Conidiomata*** on natural substrata only on base part of stroma, simple depression of surface without distinct rims, several conidiomata per stroma, but difficult to count because they fuse with neighboring ones, widely open. ***Conidial masses*** orange. ***Phialides*** not observed. No paraphyses observed. ***Conidia*** unicellular, hyaline, smooth, ventricose, with acute ends, 10–12.5 × 1.7–2.7 μm.

##### Culture characteristics.

Colonies on PDA slow-growing, attaining a diameter of 7.0–8.0 mm in 21 days at 25 °C. Colonies white to pale yellow, compact, forming a subglobose structure. Conidial masses usually abundant, sometimes forming several gushing bands, cream-colored to yellow. Colony reverse side yellowish brown, narrow white to pale yellow at the margin.

##### Habitat.

On scale insects (Coccidae, Hemiptera) and whiteflies (Aleyrodidae, Homoptera), found on the underside of dicotyledonous leaves.

##### Distribution.

China, Yunnan Province, Yuanyang County; China, Hainan Province, Changjiang County.

##### Other materials examined.

China • Yunnan Province, Yuanyang County, Xinjie Town, 23°08'N, 102°86'E, alt. 2,054 m, found on the underside of living leaves of dicotyledonous plants, 25 September 2022, Hong Yu (YHH 2209002, paratype; YFCC 22099315, ex-paratype); • Ibid., (YHH 2209003, paratype; YFCC 22099316, ex-paratype); • Ibid., (YFCC 22099317, YFCC 22099318, ex-paratype). China • Hainan Province, Changjiang County, Jianfengling National Forest Park, 18°74'N, 108°86'E, alt. 844 m, 11 March 2023, Hong Yu (YHH 2303001, YHH 2303002, YHH 2303003, YHH 2303004).

##### Notes.

Based on three-gene phylogenetic analyses, the results indicated that the three samples of *Moelleriellayuanyangensis* clustered together and formed a sister group with *M.jinghongensis*, a novel species described herein (Fig. 1). Morphologically, a comprehensive comparison could not be conducted due to the limited availability of specimens; only the anamorphic stromata of *M.yuanyangensis* and the teleomorphic stromata of *M.jinghongensis* were collected in this study. The distinguishing feature of *M.yuanyangensis* within the genus *Moelleriella* was its ventricose conidia. While species such as *M.basicystis*, *M.phyllogena*, and *M.umbospora* also produced ventricose conidia, *M.yuanyangensis* could be differentiated from these species based on its unique stromatal morphology and phylogenetic position.

#### ﻿ Moelleriella yunnanensis

Taxon classificationFungiHypocrealesClavicipitaceae

Hong Yu bis, Z.L. Yang, Z.Q. Wang & J.M. Ma sp. nov.

DB21E818-42CC-5A28-B572-35CC68D9B67A

851096

Fig. 12

##### Etymology.

Named after the location of Yunnan Province, where this species was collected.

**Figure 12.**
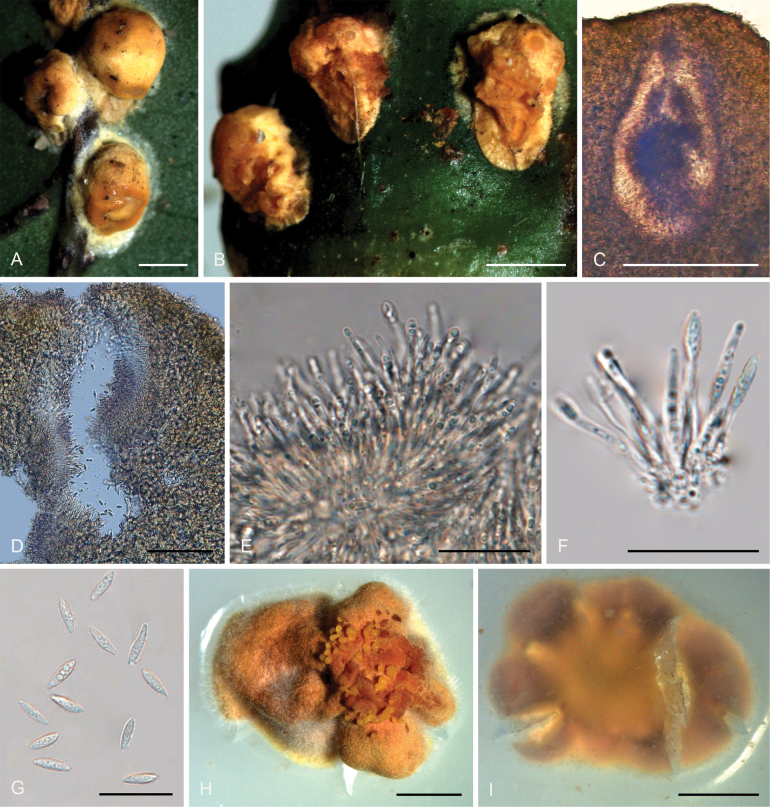
*Moelleriellayunnanensis***A, B** anamorphic stromata containing conidiomata **C, D** section of stromata showing conidiomata **E** phialides **F** phialides with conidia (arrow) at the tips **G** conidia **H** colonies obverse on PDA at 25 °C after 21 days **I** colonies reverse on PDA at 25 °C after 21 days. Scale bars: 1 mm (**A, B**); 200 µm (**C**); 100 µm (**D**); 20 µm (**E–G**); 2 mm (**H–I**).

##### Diagnosis.

*S*imilar to *Moelleriellaturbinata* and *M.epiphylla* in producing flask-shaped phialides, no paraphyses were observed, but it could be distinguished from *M.turbinata* and *M.epiphylla* by smaller conidia.

##### Type.

China • Yunnan Province, Pu’er City, Nandaohe Village, Yeyatang, 22°60'N, 100°99'E, alt. 1,000 m, found on the underside of living leaves of dicotyledonous plants, 3 August 2023, Hong Yu (YHH 2308001, holotype; YFCC 23089310, ex-type)

##### Description.

**Teleomorph**: Not known. **Anamorph: *Stromata*** on natural substrate subglobose or irregular shape, surface smooth, orange yellow to orange, 1.1–2.7 mm in diameter, sometimes with narrow hypothallus. ***Hyphae*** of stromata forming compact ***textura epidermoidea***. ***Conidiomata*** simple depressions of surface, a few conidiomata per stroma, flask-shaped, 122–315 × 65–240 μm. ***Conidial masses*** greyish yellow to orange. Phialides formed in a thick compact palisade, flask-shaped, 9.5–27 × 0.9–1.6 μm. No paraphyses were observed. ***Conidia*** unicellular, hyaline, smooth, fusoid with acute ends, straight or slightly curved, 8.8–12.5 × 2.2–3.5 μm, produced in copious slime.

##### Culture characteristics.

Colonies on PDA slow-growing, attaining a diameter of 7.0–9.0 mm in 21 days at 25 °C. Stromatic colonies yellow, orange to dark orange, surface wrinkled and tomentose. Conidial masses orange yellow, in the center of colonies. Colony reverse side orange yellow to dark brown.

##### Habitat.

On scale insects (Coccidae, Hemiptera) or whiteflies (Aleyrodidae, Homoptera), generally on the abaxial and adaxial underside of living leaves, and sometimes on twigs, of dicotyledonous plants.

##### Distribution.

China, Yunnan Province, Pu’er City.

##### Other materials examined.

China • Yunnan Province, Pu’er City, Nandaohe Village, Yeyatang, 22°60'N, 100°99'E, alt. 1,000 m, found on the underside of living leaves of dicotyledonous plants, 3 August 2023, Hong Yu (YHH 2308002, paratype; YFCC 23089311, ex-paratype); • Ibid., (YHH 2308003, YHH 2308004, YHH 2308005, YHH 2308006, YHH 2308007).

##### Notes.

Phylogenetic analyses indicated that the two samples of *Moelleriellayunnanensis* clustered together, forming a distinct clade within the Globose clade of *Moelleriella*. This clade was closely associated with *M.turbinata* and *M.epiphylla* (Fig. 1). Morphologically, *M.yunnanensis* shared similarities with *M.turbinata* and *M.epiphylla*, characterized by flask-shaped phialides, abundant conidial production in mucilage, and the absence of paraphyses (Chaverri et al. 2008). Ecologically, these three species were commonly found on both the abaxial and adaxial surfaces of leaves as well as on twigs (Chaverri et al. 2008). However, *M.yunnanensis* could be differentiated from *M.turbinata* by the color and shape of the stroma, which exhibited greyish-yellow to orange conidial masses, flask-shaped conidiomata, and fusiform, smaller conidia (Chaverri et al. 2008). Additionally, it differed from *M.epiphylla* by its flask-shaped conidiomata, narrower phialides, and fusiform, smaller conidia (Chaverri et al. 2008).

#### ﻿ Paramoelleriella

Taxon classificationFungiHypocrealesClavicipitaceae

Hong Yu bis, Z.L. Yang, Z.Q. Wang & Jing Zhao gen. nov.

6CDC025D-E48C-544B-980C-B86C32691FCD

851097

##### Etymology.

Refers to its close morphological relationship to *Moelleriella*.

##### Type species.

*Paramoelleriellacurvospora* Hong Yu bis, Z.L. Yang, Z.Q. Wang & Jing Zhao.

##### Description.

**Teleomorph: *Stromata*** globose to subglobose, yellow to orange. ***Perithecia*** densely arranged in stromata, completely embedded, flask-shaped, numerous perithecia per stroma. ***Ostioles*** deep orange to dark brown. ***Asci*** cylindrical, ascospores disarticulating into part-spores that were short-cylindrical with rounded ends. **Anamorph: *Stromata***, orange red. ***Conidioma*** large, few conidiomata per stroma, widely open orifice. In section, the conidioma irregular-shaped. ***Conidia*** unicellular, fusoid with acute ends, but curved to one side. ***Paraphyses*** present.

##### Habitat.

On scale insects (Coccidae, Hemiptera) or whiteflies (Aleyrodidae, Homoptera), found on the underside of dicotyledonous leaves.

##### Notes.

The genera *Conoideocrella*, *Dussiella*, *Helicocollum*, *Hyperdermium*, *Hypocrella*, *Moelleriella*, *Orbiocrella*, *Regiocrella*, and *Samuelsia* have been reported as scale insects and whiteflies pathogenic fungi in Clavicipitaceae (Sullivan et al. 2000; Chaverri et al. 2005a, b, 2008; Johnson et al. 2009; Luangsa-ard et al. 2017). In this study, phylogenetic analyses showed that the new genus *Paramoelleriella*, pathogenic on scale insects, was proposed according to the type species *P.curvospora* phylogenetic placement and was closely related to *Hypocrella*, *Samuelsia*, and *Moelleriella*. Morphologically, *Paramoelleriella* was similar to *Moelleriella* by its shape and color of stromata, filiform multi-septate ascospores disarticulated into part-spores, and fusoid conidia. However, it differed from *Moelleriella* by producing conidia that curve to one side, and it could be distinguished from *Hypocrella* and *Samuelsia* by its ascospores disarticulating into part-spores, while the latter two did not.

#### ﻿ Paramoelleriella curvospora

Taxon classificationFungiHypocrealesClavicipitaceae

Hong Yu bis, Z.L. Yang, Z.Q. Wang & Jing Zhao sp. nov.

C329FBD0-7D10-58EA-9841-F2C96CCCF8B8

851098

Fig. 13

##### Etymology.

*curvospora*, indicating that the conidia were curved.

##### Diagnosis.

Similar to *Moelleriellaglobostromata* in color and shape of stromata, but it could be distinguished from *M.globostromata* by its smaller stromata, larger perithecia, and larger asci.

**Figure 13.**
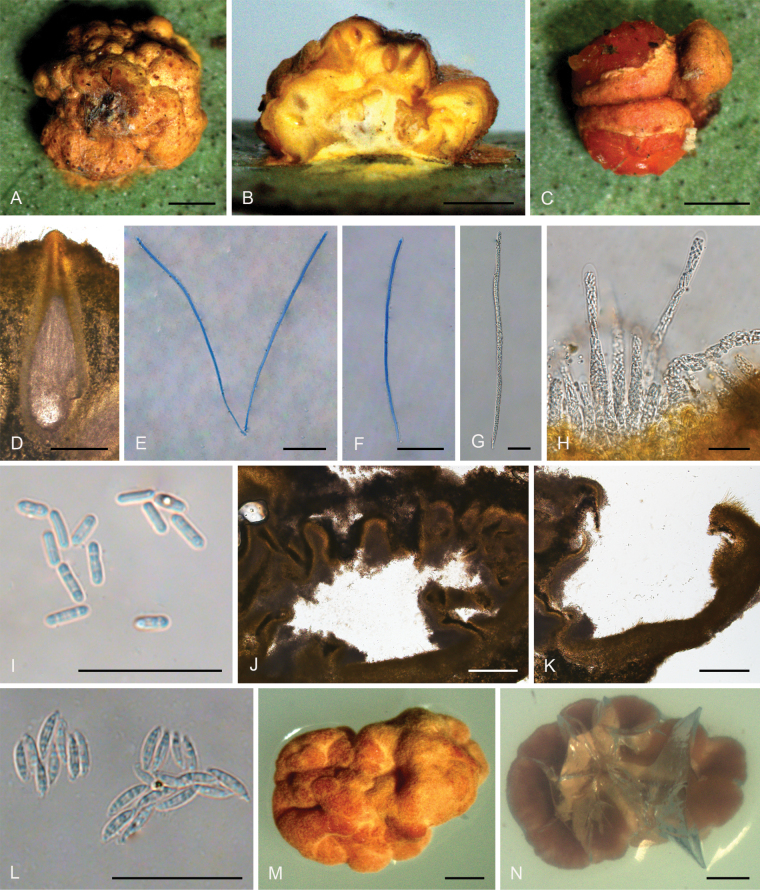
*Paramoelleriellacurvospora***A, B** teleomorphic stromata containing perithecia **C** anamorphic stroma containing conidiomata **D** perithecia **E–H** mature asci with developing asci **I** part-spores **J, K** section of stroma showing conidiomata **L** conidia **M** colonies obverse on PDA at 25 °C after 21 days **N** colonies reverse on PDA at 25 °C after 21 days. Scale bars: 1 mm (**A–C**); 200 µm (**D**); 50 µm (**E–G**); 20 µm (**H–I**); 200 µm (**J–K**); 20 µm (**L**); 1 mm (**M–N**).

##### Type.

China • Yunnan Province, Jinping County, the Wutai Mountains, 22°76'N, 103°48'E, alt. 1,544 m, found on the underside of living leaves of dicotyledonous plants, 26 May 2023, Hong Yu (YHH 2305001, holotype).

##### Description.

**Teleomorph: *Stromata*** globose to subglobose, uneven surface, constricted at base, 3.8–3.9 mm in diameter, 1.8–1.9 mm high, pale orange, opaque, with a narrow hypothallus. ***Hyphae*** of stromata forming compact ***textura intricata*** to ***epidemoidea***. ***Perithecia*** densely arranged in stromata, completely embedded, flask-shaped, numerous perithecia per stroma (>70), 345–745 × 130–235 μm. ***Ostioles*** deep orange to dark brown. ***Asci*** cylindrical, 190–370 × 4.2–10.2 μm, caps 2.8–4.5 μm thick. ***Ascospores*** initially filiform, disarticulating into part-spores that were short-cylindrical with rounded ends, 4.7–7.3 × 1.1–1.9 μm. **Anamorph**: (The teleomorph and anamorph are not found in the same stroma.) ***Stromata***, orange red, 2.0–3.0 mm in diameter. ***Conidioma*** large, few conidiomata per stroma, widely open orifice. In section, the conidioma irregular-shaped. ***Conidia*** hyaline, smooth, unicellular, fusoid with acute ends, but curve to one side, 6.6–9.2 × 1.5–2.1 μm. ***Paraphyses*** present, linear, filiform, up to 133 µm long.

##### Habitat.

On scale insects (Coccidae, Hemiptera) or whiteflies (Aleyrodidae, Homoptera), found on the underside of dicotyledonous leaves.

##### Distribution.

China, Yunnan, Jinping County.

##### Other materials examined.

China • Yunnan Province, Jinping County, the Wutai Mountains, 22°76'N, 103°48'E, alt. 1,544 m, found on the underside of living leaves of dicotyledonous plants, 26 May 2023, Hong Yu (YHH 2305002, paratype).

##### Notes.

Phylogenetically, *Paramoelleriellacurvospora* formed a separate clade and was closely related to *Hypocrella*, *Samuelsia*, and *Moelleriella*, with high statistical support from BI (PP = 91%) and ML (BP = 90%) (Fig. 1). Morphologically, the teleomorph of *P.curvospora* was similar to that of *M.globostromata* in color and shape of stromata, and both had completely embedded, flask-shaped perithecia and short-cylindrical part-spores. However, it could be distinguished from *M.globostromata* by its smaller stromata, larger perithecia (345–745 × 130–235 vs. 110–410 × 110–205), larger asci (190–370 × 4.2–10.2 vs. 85–170 × 3.0–6.3), and paraphyses present.

#### ﻿ Polymicrospora

Taxon classificationFungiHypocrealesClavicipitaceae

Hong Yu bis, Z.Q. Wang, Z.L. Yang & J.M. Ma gen. nov.

8E1BD857-14F9-5A4E-B595-6B733A39572B

851099

##### Etymology.

*Polymicrospora*, “*Poly*” indicates that the number of part-spores from the mature asci is very much, “*micro*” means part-spores were very small.

##### Type species.

*Polymicrosporacaiyangheensis* Hong Yu bis, Z.Q. Wang, Z.L. Yang & J.M. Ma.

##### Description.

**Teleomorph: *Stromata*** usually thin pulvinate, snow white to off-white, surface smooth in the middle. Many perithecia in mature stromata. ***Perithecia*** densely arranged in stroma, semi-embedded, obpyriform, or oval. ***Ostioles*** with slightly convex, reddish brown to dark brown. ***Asci*** cylindrical. ***Ascospores*** initially filiform, disarticulating into part-spores. ***Part-spores*** oval, small, large amount. **Anamorph**: Not known.

##### Habitat.

Found on the adaxial or underside of living dicotyledonous and fern leaves.

##### Notes.

The host of the genus *Polymicrospora* was unknown, and in this study, species of the genus *Polymicrospora* were collected in the course of investigating the resources of the genus *Hypocrellas. lato.*, during which we found that species of the genus *Polymicrospora* were not as densely distributed as species of the genus *Hypocrellas. lato.* (i.e., multiple stromata may be collected from a single leaf or from a single tree). Species of the genus *Polymicrospora* were uncommon; usually, two or three stromata were collected in a survey area, and there was usually only one stroma on a leaf. No scale insects or whiteflies were found on the leaves from which the stromata of the genus was collected or on the whole tree. The stromata were also not found to contain any insects under the laboratory microscope, so the host could not be identified in this study. Species of this genus were characterized by white stroma; the surface of the stroma was smooth; and the perithecia were densely distributed on the mature stroma. These characteristics allowed us to almost certainly identify it as a species of this genus during the field collection.

#### ﻿ Polymicrospora caiyangheensis

Taxon classificationFungiHypocrealesClavicipitaceae

Hong Yu bis, Z.Q. Wang, Z.L. Yang & J.M. Ma sp. nov.

64429E93-5A93-51B8-87D8-881B34EE2611

851100

Fig. 14

##### Etymology.

Named after the location of the holotype collection—in order to commemorate the Sun River’s former name, Caiyang River.

**Figure 14.**
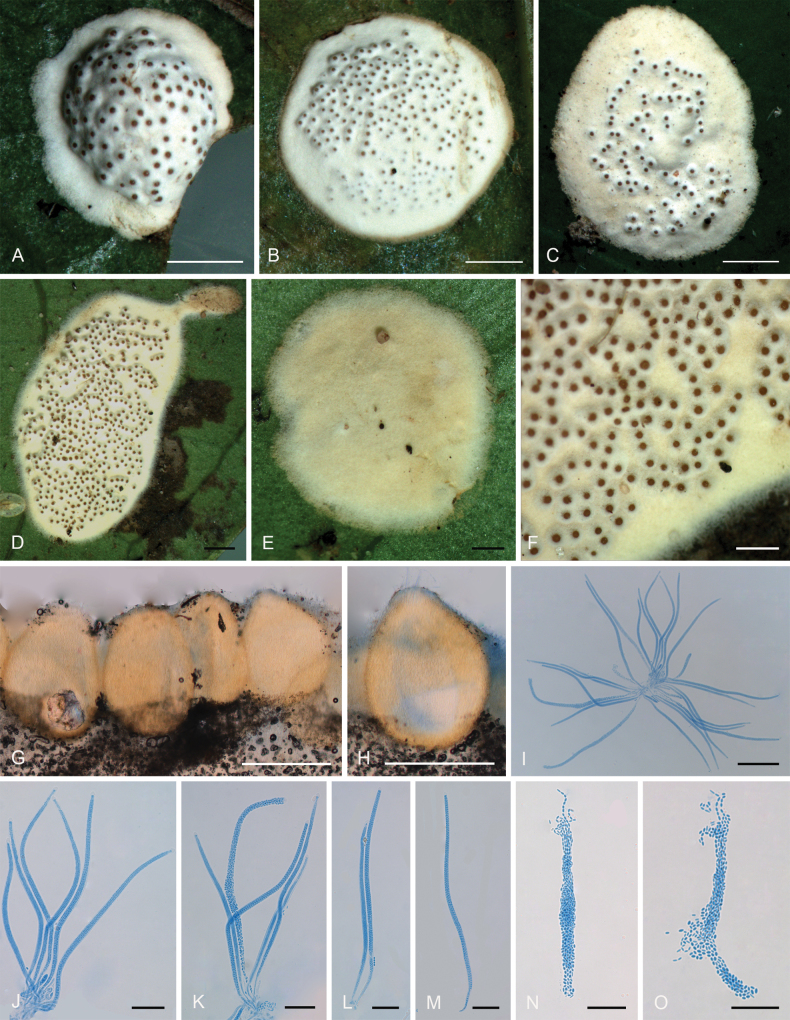
*Polymicrosporacaiyangheensis***A–F** teleomorphic stromata containing perithecia **G–H** perithecia **I–M** mature asci with developing asci **N, O** asci and part-spores. Scale bars: 1 mm (**A–C**); 2 mm (**D–E**); 1 mm (**F**); 200 µm (**G–H**); 50 µm (**I**); 20 µm (**J–O**).

##### Type.

China • Yunnan Province, Pu’er City, Simao District, Sun River National Forest Park, N 22°62′, E 101°13′, alt. 1,336 m, found on the underside of living leaves of ferns, 27 September 2023, Hong Yu (YHH 2309001, holotype).

##### Description.

**Teleomorph: *Stromata*** usually thin pulvinate, oviform to elongated ellipsoid, occasionally with a hemispherical bulge in the center, snow white to off-white, surface smooth in middle and tomentose in periphery, 1.5–9.1 mm in diameter. Many perithecia in mature stromata. ***Perithecia*** densely arranged in stroma, semi-embedded, obpyriform or oval, 241–369 × 196–308 μm. ***Ostioles*** with slightly convex, reddish brown to dark brown. ***Asci*** cylindrical, 104–198 × 1.9–4.0 μm, caps 1.4–2.9 μm thick. ***Ascospores*** initially filiform, disarticulating into part-spores. ***Part-spores*** oval, small, large amount, 1.4–2.4 × 0.5–1.3 μm. **Anamorph**: Not known.

##### Habitat.

Found on the underside of living dicotyledonous leaves and ferns.

##### Distribution.

China, Yunnan Province, Pu’er City, and Jinghong City.

##### Other materials examined.

China • Yunnan Province, Pu’er City, Simao District, Sun River National Forest Park, N 22°62′, E 101°13′, alt. 1,336 m, found on the underside of living leaves of dicotyledonous plants and ferns, 27 September 2023, Hong Yu (YHH 2309006, YHH 2309007, YHH 2309008). China • Yunnan Province, Pu’er City, Simao District, Xinfang Reservoir, N 22°71′, E 100°95′, alt. 1,329 m, found on the underside of living leaves of dicotyledonous plants, 6 October 2019, Hong Yu (YHH 1906002, YHH 1906003). China • Yunnan Province, Jinghong City, Puwen Town, 22°52'N, 100°98'E, alt. 1,085 m, found on the underside of living leaves of dicotyledonous plants, 26 September 2023, Hong Yu (YHH 2309004, YHH 2309005).

##### Notes.

Regarding its phylogenetic position, this species formed a sister clade to the species of *Collarinaaurantiaca* (Fig. 1). However, its macromorphological and micromorphological characteristics differed from those of *C.aurantiaca* (Crous et al. 2014). Based on the habitats from which the species was collected and its macroscopic morphology, it exhibited significant similarities to the genus *Hypocrellas. lato.* Nevertheless, it could be differentiated from the genera *Hypocrellas. str.* and *Samuelsia* by the disarticulation of its ascospores into part-spores, a feature absent in the latter two genera. Additionally, it could be distinguished from the genera *Moelleriella* and *Paramoelleriella* by smaller, oval-shaped part-spores.

## ﻿Discussion

This research concentrated on the phylogenetic investigation of whitefly parasitic fungi from Yunnan and Hainan Provinces, China. Based on phylogenetic analysis, two novel genera and thirteen new species within the family Clavicipitaceae were introduced. The phylogenetic tree depicted that the genus *Moelleriella* diverged into two principal clades, namely the Effuse clade and the Globose clade. Notably, the Effuse clade further diverged into two sister subclades, designated as subclade I and subclade II (Fig. 1). Additionally, the Pulvinate clade comprised *Hypocrellas. str.* (Pulvinate clade A) and *Samuelsia* (Pulvinate clade B). The newly described genus *Paramoelleriella* exhibited proximity to both the Pulvinate clade A and B, branching off independently. Furthermore, the genus *Polymicrospora* formed a sister clade with *Collarina* (Fig. 1).

Different branches had different morphological features, but these features are not unique to each group and can be found overlapping with crossovers between evolutionary branches in *Moelleriella* (Chaverri et al. 2008; Khonsanit et al. 2021). The subclade I contained three new species, namely *M.multiperitheciata*, *M.puwenensis*, and *M.qionzhongensis*, and thirteen known species. Among them, *M.chiangmaiensis*, *M.flava*, *M.mollii*, *M.multiperitheciata*, *M.nanensis*, *M.nivea*, *M.ochracea*, *M.phukhiaoensis*, *M.puwenensis*, *M.sinensis*, and *M.zhongdongii* were described on the basis of the anamorphic and teleomorphic states. *Moelleriellagracilispora*, *M.kanchanaburiensis*, *M.madidiensis*, *M.pongdueatensis*, and *M.qionzhongensis* were described only based on the anamorphic state. The species in this clade, with the exception of *M.madidiensis*, *M.ochracea*, and *M.zhongdongii* from the New World, are all from the Old World (Chaverri et al. 2008; Li et al. 2016; Chen et al. 2020; Yuan et al. 2020; Khonsanit et al. 2021). They have obpyriform or flask-shaped perithecia, fusiform conidia (with the exception of narrowly cylindrical in *M.phukhiaoensis*), and present paraphyses (with the exception of *M.madidiensis*). The subclade II contained four new species, all from the Old World, namely *M.jinghongensis*, *M.hainanensis*, *M.pseudothanathonensis*, and *M.yuanyangensis*. Six of the thirteen known species were from the Old World, namely *M.alba*, *M.chumphonensis*, *M.puerensis*, *M.raciborskii*, *M.simaoensis*, and *M.thanathonensis*, and seven species were from the New World, namely, *M.basicystis*, *M.disjuncta*, *M.evansii*, *M.libera*, *M.phyllogena*, *M.rhombispora*, and *M.umbospora* (Chaverri et al. 2008; Mongkolsamrit et al. 2015; Tibpromma et al. 2017; Wang et al. 2022; Yang et al. 2023). Except for *M.pseudothanathonensis*, *M.yuanyangensis*, and *M.thanathonensis*, which were described based on anamorphic states, and *M.jinghongensis* and *M.hainanensis*, which were described based on teleomorphic states, the rest of the species were described based on both anamorphic and teleomorphic states. The subclade II species mostly have flask-shaped perithecia, and some species have other shapes, for example, narrowly ovoids in *M.hainanensis*; subglobose to ovoid in *M.evansii* and *M.puerensis*; globose to ovoid in *M.libera* and *M.raciborskii*; and obpyriform in *M.jinghongensis*. Some species had absent paraphyses, except *M.simaoensis*, *M.puerensis*, *M.thanathonensis*, *M.chumphonensis*, *M.libera*, *M.rhombispora*, *M.raciborskii*, and *M.pseudothanathonensis*. And most species had fusiform conidia, except for ventricose conidia in *M.basicystis*, *M.phyllogena*, *M.umbospora*, and *M.yuanyangensis*. The Globose clade had two new species from the Old World, namely *M.globostromata* and *M.yunnanensis*, and eight known species, except *M.africana*, *M.insperata*, and *M.schizostachyi*, which are from the Old World; the rest (*M.boliviensis*, *M.epiphylla*, *M.macrostroma*, *M.sloaneae*, and *M.turbinata*) are from the New World (Chaverri et al. 2008). The Globose group species have globose stromata that are generally darker in color, large (except *M.yunnanensis*), compact, hard or coriaceous, and without hypothalli (except *M.yunnanensis*). *Moelleriellasloaneae* is morphologically different from other species in the group. The Pulvinate clade A had two new species (*H.limushanensis* and *H.yunnanensis*) from the Old World and seven known species; among them, three (*H.calendulina*, H.cf.discoidea, and *H.discoidea*) were from the Old World and four (*H.citrina*, *H.disciformis*, *H.hirsuta*, and *H.viridans*) came from the New World (Hywel-Jones and Evans 1993; Chaverri et al. 2008; Mongkolsamrit et al. 2009). The Pulvinate clade A comprised the species that have pulvinate or cushion-like stromata, flask-shaped perithecia (except for *H.citrina*, which is subglobose), fusoid conidia (except *H.discoidea*), and paraphyses present.

The species *Hypocrella*, *Samuelsia*, *Moelleriella*, and *Paramoelleriella* are parasitic on whiteflies and scale insects. Before the stromata structures become visible, the insect host is almost always fully devoured by the fungus, at which point it is nearly impossible to identify the insect (Evans 1988; Meekes et al. 1994; Chaverri et al. 2008). As a result, there is little information available on *Hypocrella*, *Samuelsia*, *Moelleriella*, and *Paramoelleriella* host specificities (Chaverri et al. 2008). Both whiteflies and scale insects have a wide variety of species, are widely distributed, and are harmful to a large number of plants. Among them, whiteflies are a group with a worldwide distribution of 161 genera and 1,556 species, including the subfamilies Aleurodicinae, Aleyrodinae, and Udamoselinae (Mound and Halsey 1978; Martin and Mound 2007; Lourenção et al. 2022). The subfamily Aleurodicinae is mainly found in the New World, whereas the subfamily Aleyrodinae has a worldwide distribution (Lourenção et al. 2022). They attack crops and forest plants, causing damage that is hard to estimate due to the sheer number of crops they attack, their incredibly wide geographic distribution, and, in particular, their capacity to spread viruses, which cause much more damage than insects do (Song et al. 2011; Xiong et al. 2011; Wang et al. 2014; Knapp et al. 2020). The morphological identification of Aleyrodidae was done using the pupal case (Martin et al. 1987). Similar to whiteflies, scale insects are ubiquitous (they are found on every continent except Antarctica), most are host-plant specialists and are difficult to detect and highly invasive, and many species are serious agricultural pests that damage plants through sap loss, promote the growth of sooty moulds, and transmit plant diseases (Wang et al. 2001; Ouvrard et al. 2013; Morales et al. 2016; Arias-Corpuz et al. 2021; Lu et al. 2023). Currently, there are at least 8,194 described species, classified into 50 families (Morales et al. 2016). Scale insects superficially may be very similar to closely related species, and their species identification is determined by examining the morphological characteristics of adult females, which necessitates the preservation of the adult cuticle, proper specimen preparation, and examination by a qualified taxonomist, so identification is still difficult for scale insects (Gullan and Kosztarab 1997; Deng et al. 2012; Ouvrard et al. 2013). The identification of host species for *Hypocrella*, *Samuelsia*, *Moelleriella*, and *Paramoelleriella* is difficult due to limitations in the developmental stages of whiteflies and scale insects, as well as a lack of expertise in insect identification. Although it can be found that there are uninfected whiteflies or scale insects at the time of specimen collection, it is not sure whether they are the same species as the infected insects because, in many cases, several species of scale insects or whiteflies are found on the same leaf (Petch 1921), and they are not necessarily at the developmental stage used for morphological identification (e.g., whitefly is the pupal case stage and scale is the adult female), so they cannot be identified by morphology. In addition, amplifying the insect gene by extracting the entire stromata with the Genomic DNA Purification Kit (Qiagen GmbH, Hilden, Germany) was tried in this study, but all failed. Although more comprehensive survey studies have been conducted by previous researchers (Chaverri et al. 2008; Mongkolsamrit et al. 2015; Li et al. 2016; Tibpromma et al. 2017; Chen et al. 2020; Yuan et al. 2020; Khonsanit et al. 2021; Wang et al. 2022; Yang et al. 2023), surveys of fungal biodiversity in other under-explored areas may reveal more undescribed species due to the species diversity of the host scale insects or whiteflies and their wide distribution.

Species within the genus *Hypocrellas. lato.* have been documented across 12 provinces (Fujian, Guangdong, Guangxi, Guizhou, Hainan, Hubei, Hunan, Jiangxi, Shanxi, Sichuan, Yunnan, and Zhejiang) and Taiwan, with the majority exhibiting distribution patterns in tropical, subtropical, and temperate regions (Ma 2008; Wang 2009; Qiu 2013; Chen et al. 2020; Yuan et al. 2020; Wang et al. 2022; Yang et al. 2023). However, the reliance on morphological characteristics for species identification in prior studies, due to insufficient utilization of molecular data, has potentially resulted in misidentifications. Given that these species serve as natural regulators of whitefly and scale insect populations, and considering the advantages of biological control over chemical methods—such as environmental safety, sustained pest suppression, and the absence of resistance development—the discovery of additional species contributes valuable scientific insights for the conservation and sustainable utilization of microbial and fungal resources.

## Supplementary Material

XML Treatment for Hypocrella limushanensis

XML Treatment for Hypocrella yunnanensis

XML Treatment for Moelleriella globostromata

XML Treatment for Moelleriella hainanensis

XML Treatment for Moelleriella jinghongensis

XML Treatment for Moelleriella multiperitheciata

XML Treatment for Moelleriella pseudothanathonensis

XML Treatment for Moelleriella puwenensis

XML Treatment for Moelleriella qionzhongensis

XML Treatment for Moelleriella yuanyangensis

XML Treatment for Moelleriella yunnanensis

XML Treatment for Paramoelleriella

XML Treatment for Paramoelleriella curvospora

XML Treatment for Polymicrospora

XML Treatment for Polymicrospora caiyangheensis

## ﻿Additional information

### Author ORCIDs

Jin-Mei Ma https://orcid.org/0009-0008-9440-6947

Hong Yu https://orcid.org/0000-0002-2149-5714

### Data availability

All of the data that support the findings of this study are available in the main text.
